# Intra- and inter-hemispheric processing during binocular rivalry in mild glaucoma

**DOI:** 10.1371/journal.pone.0229168

**Published:** 2020-02-25

**Authors:** Luminita Tarita-Nistor, Saba Samet, Graham E. Trope, Esther G. González

**Affiliations:** 1 Krembil Research Institute, Toronto Western Hospital, Toronto, Ontario, Canada; 2 Faculty of Medicine, University of Toronto, Toronto, Ontario, Canada; 3 Ophthalmology and Vision Sciences, University of Toronto, Toronto, Ontario, Canada; LV Prasad Eye Institute, INDIA

## Abstract

Glaucoma is considered a progressive optic neuropathy because of the damage and death of the retinal ganglion cells. It is also a neurodegenerative disease because it affects neural structures in the visual system and beyond, including the corpus callosum–the largest white matter structure involved in inter-hemispheric transfer of information. In this study we probed the dysfunction of the inter-hemispheric processing in patients with mild glaucoma using the phenomenon of binocular rivalry. Patients with mild glaucoma and no measurable visual field defects and age-matched controls underwent a thorough visual assessment. Then they participated in a series of psychophysical tests designed to examine the binocular rivalry derived from intra- and inter-hemispheric processing. Static horizontal and vertical sinewave gratings were presented dichoptically using a double-mirror stereoscope in 3 locations: centrally, to probe inter-hemispheric processing, and peripherally to the left or to the right, to probe intra-hemispheric processing. Although the two groups were matched in functional measures, rivalry rate of the glaucoma group was significantly lower than that of the control group for the central location, but not for the peripheral location. These results were driven mainly by the patients with normal tension glaucoma whose average rivalry rate for the central location (from which information reaches the two hemispheres) was almost half (46% lower) that of the controls. These results indicate a dysfunction in inter-hemispheric transfer in mild glaucoma that can be detected behaviourally before any changes in standard functional measures.

## Introduction

Glaucoma is the second leading cause of blindness affecting more than 60 million people worldwide.[1] It is considered a progressive optic neuropathy because of the damage and death of the retinal ganglion cells (RGCs),[2] as well as a neurodegenerative disease because it affects neural structures in the visual system far beyond the retina.[3] Histopathological examination of the brain from patients with advanced glaucoma has revealed degeneration of the intracranial optic nerve, the lateral geniculate nucleus, and the visual cortex,[4] while neuroimaging studies have shown changes in the entire primary visual pathways (i.e., retina, optic nerve, optic chiasm, optic tract, lateral geniculate nucleus, optic radiation, and the visual cortex).[5–10] The degeneration observed in the visual pathways may be caused by the propagation of the pre-geniculate damage, through mechanisms such as Wallerian degeneration.[3,9]

Glaucoma is also associated with neurodegeneration beyond the primary visual pathways, including in the corpus callosum [9,10] which is the most important brain structure involved in inter-hemispheric transfer of visual information.[11] It has been found that, compared to controls, the volume of the corpus callosum is increased in early glaucoma, and decreased in more advanced stages of disease.[10] Boucard et al.[9] also found neurodegeneration in the body and splenium of the corpus callosum in patients with moderate-stage glaucoma, and proposed that this callosal degeneration could not be explained by the propagation of the pre-geniculate damage but rather by an additional degenerative mechanism. However, we do not have any behavioural evidence that dysfunction in inter-hemispheric transfer—suggestive of callosal degeneration—exists in these patients. It is known that a large proportion of RGCs is lost before any visual field defects are detected with standard perimetry [12] making glaucoma notoriously difficult to detect. A behavioural test targeting inter-hemispheric transfer could serve as a biomarker for early detection.

A potential non-invasive psychophysical test to probe inter-hemispheric transfer integrity involves the phenomenon of binocular rivalry. In binocular rivalry the perceptual experience alternates between the information from each eye despite unchanging visual stimuli. When one image is presented to one eye and a different image is presented to the other eye in a region of retinal correspondence, the brain cannot integrate the two into a stable percept. Rather, the two images compete for visual awareness, with one image being perceived while the other is suppressed, only to reverse the visual dominance moments later. Binocular rivalry provides insights into the dynamics of the visual system, including the intra- and inter-hemispheric processing of visual information,[13–18] but despite its long history this phenomenon is not fully understood. Rivalry processing depends on the complexity of the stimuli [15,18,19] and it has been proposed that a unified model of rivalry could involve multi-level hierarchic rivalry stages, with rivalry of traditional stimuli (e.g., static orthogonal sinewave gratings) being processed in the earliest stages of the visual hierarchy.[20]

**Fig 1:**
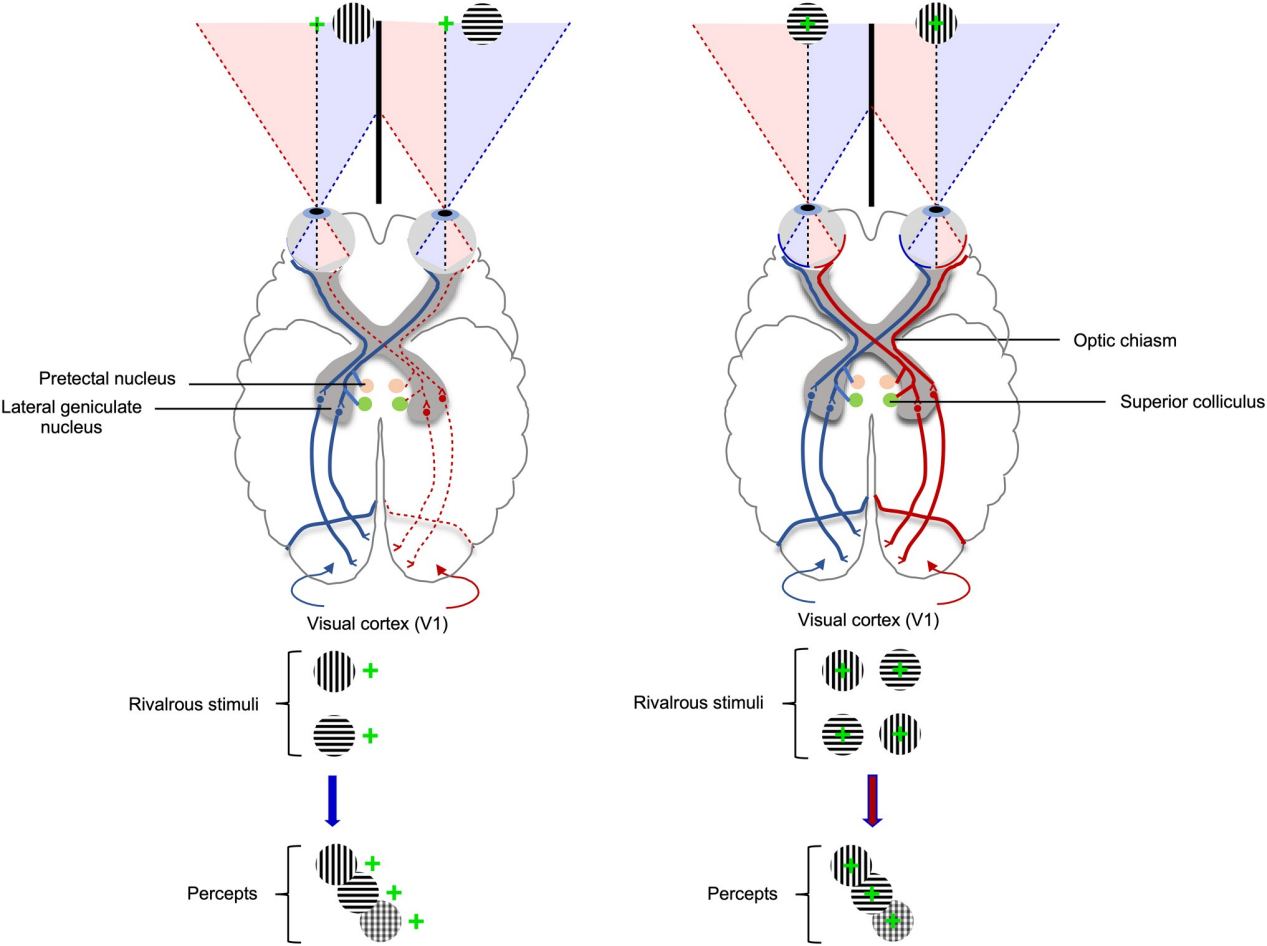
Schematic of the visual pathways during binocular rivalry processing. Left panel shows that when two different stimuli are presented dichoptically (through a double-mirror stereoscope, not shown on this schematic) on the right hemifield, both stimuli reach the contralateral hemisphere eliciting only intra-hemispheric processing. The right panel shows that when the same stimuli are presented dichoptically in a central location, both stimuli reach both hemispheres and rivalry processing needs to be synchronized through inter-hemispheric transfer.

Under natural binocular viewing conditions, visual information from the left half of the visual field is projected initially to the right occipital lobe and that from the right half of the visual field to the left occipital lobe. This is possible because the primary visual pathway is organized in such a way that the optic nerve fibers from the nasal hemiretina of one eye cross at the optic chiasm and come together with the uncrossed fibers from the temporal hemiretina of the other eye to form the optic tract. The information from both hemispheres needs to be integrated for a coherent visual percept, and the most important brain structure involved in inter-hemispheric transfer is the corpus callosum.[11] During binocular rivalry, when two traditional stimuli are presented dichoptically in a peripheral location on the left or on the right, visual information about both stimuli reaches the contralateral hemisphere (as shown in the schematics from Fig 1, left panel) and rivalry relies only on intra-hemispheric processing. Conversely, when two traditional stimuli are presented dichoptically in a central location, visual information about both stimuli reaches both hemispheres (see Fig 1, right panel), and the rivalry processing is combined and synchronized through inter-hemispheric communication. Rare patients who have undergone corpus callosotomy show normal rivalry processes when stimuli are presented only on the right or on the left hemifield, but not when presented centrally. [21,22]

The purpose of this study was to probe the integrity of the inter-hemispheric transfer using binocular rivalry in patients with mild glaucoma and no detectable deficits on standard functional measures. Static horizontal and vertical sinewave gratings were shown dichoptically using a double-mirror stereoscope in 3 locations: centrally, to test inter-hemispheric processing, and peripherally on the left or on the right, to test intra-hemispheric processing. Based on imaging findings of callosal degeneration in patients with glaucoma, [9,10] we hypothesized a dysfunction of the inter-hemispheric transfer that could be detected behaviourally in the early stages of the disease.

## Materials and methods

### Participants

Thirty-one patients with mild open angle glaucoma (mean age 66 ± 12 years) and 30 age-matched controls (mean age 63 ± 10 years) participated in this study. A diagnosis of mild glaucoma was made by an experienced glaucoma specialist (the author GET) and was based on 1) changes consistent with the diagnosis of mild glaucoma shown by consecutive clinical examinations of the status of the optic disc, 2) intraocular pressure level, and/or 3) a family history of glaucoma. Mild glaucoma corresponded to stage 0 to 1 of the Hodapp-Parrish Anderson Glaucoma Grading Scale.

#### Inclusion criteria

Care was taken to select participants—both patients and controls—with equivalent structural and functional measures in the two eyes. For the glaucoma group, inclusion criteria included: 1) confirmed diagnosis of mild bilateral open angle glaucoma; 2) no other comorbid ocular pathologies; 3) no significant functional or structural asymmetries between the two eyes; and 4) no significant monocular or binocular functional deficits (i.e., normal or close to normal visual acuity, stereo acuity, visual field’s mean deviation). Patients were also included if they had intraocular pressure normalized with medication (i.e., below 21mmHg). Age-matched controls were included if they had 1) no ocular pathologies, and 2) no functional and structural deficits or asymmetries in the two eyes.

#### Exclusion criteria

Patients with more advanced stages of glaucoma and/or a history of eye surgery were excluded. Patients and age-matched control participants with cognitive impairment, a history of neurological disease, or other significant ocular diseases with the exception of symmetric mild cataracts were not included in this study. Participants who were not able to fuse the fixation crosses during the rivalry experiment were also excluded.

Twenty patients had a diagnosis of primary open angle glaucoma (POAG) and 11 had normal tension glaucoma (NTG). All patients were under treatment and/or monitored for disease progression by a glaucoma specialist at the Eye Clinic from the Toronto Western Hospital. The control participants were volunteers or recruited from advertisements throughout the hospital. Testing was performed at the Ocular Motor Laboratory at the same hospital. Written informed consent was obtained from all participants after the study was explained in detail. Ethics approval was obtained from the University Health Network’s Research Ethics Board and the research was conducted in accordance with the tenets of the declaration of Helsinki. Demographics and clinical characteristics of the two groups are presented in Table 1. Detailed analysis of the functional and structural measures for the two groups is presented in the Results section.

**Table 1:** Demographic and clinical characteristics for the glaucoma and control groups, along with p values for the independent-sample t-tests.

	Glaucoma Group	Control Group	p value
N [M/F]	31 [19/12]	30 [18/12]	-
Age (years)	66 ± 12	63 ± 10	0.24
Stereo acuity (sec)	100 ± 187	46 ± 75	0.15
Visual acuity 96% contrast (logMAR)			
Binocular	-0.05 ± 0.08	-0.14 ± 0.13	***0*.*004***
Right eye	-0.02 ± 0.11	-0.11 ± 0.13	***0*.*005***
Left eye	0.00 ± 0.10	-0.10 ± 0.11	***0*.*001***
Visual acuity 25% contrast (logMAR)			
Binocular	0.06 ± 0.12	-0.03 ± 0.11	***0*.*004***
Right eye	0.10 ± 0.13	0.00 ± 0.12	***0*.*003***
Left eye	0.12 ± 0.14	0.03 ± 0.12	***0*.*009***
Visual field mean deviation (dB)			
Right eye	-0.18 ± 1.63	0.62 ± 1.82	0.08
Left eye	-0.36 ± 1.70	0.27 ± 1.67	0.16
Retinal nerve fiber layer (μm)			
Right eye	80.8 ± 10.1	89.6 ± 9.3	***0*.*001***
Left eye	78.7 ± 8.2	88.0 ± 10.4	***0*.*000***
Average cup-to-disc ratio			
Right eye	0.68 ± 0.07	0.49 ± 0.13	***0*.*000***
Left eye	0.68 ± 0.07	0.48 ± 0.12	***0*.*000***
Vertical cup-to-disc ratio			
Right eye	0.67 ± 0.08	0.47 ± 0.11	***0*.*000***
Left eye	0.68 ± 0.09	0.47 ± 0.13	***0*.*000***
MOCA cognitive test	27.6 ± 1.6	28.1 ± 1.9	0.38

### Apparatus and stimuli

Functional, structural, and psychophysical measures were obtained. Functional measures were visual acuity, stereo acuity, and visual field’s sensitivity. Structural measures were average cup-to-disc ratio, vertical cup-to-disc ratio, and peripapillary retinal nerve fiber layer (RNFL) thickness. Psychophysical measures were obtained from binocular rivalry tests and included rivalry rate and proportion of percept dominance (i.e., horizontal, vertical, or mixed). In addition, cognitive abilities were tested with the Montreal Cognitive Assessment test (MoCA, www.mocatest.org).

#### Functional measures

*Visual acuity*. Visual acuity was measured at a distance of 6 m with a computerized version of the ETDRS (Early Treatment Diabetic Retinopathy Study) chart (single line) using the Accommodata Stimuli System, Version 3.5 (Haag–Streit, Mason, OH). Measurements were performed binocularly and monocularly for each eye, with the participant’s habitual corrective spectacles, at high (95%) and low (25%) contrast. A letter-by-letter scoring system was used.

*Stereo acuity*. Stereo acuity was assessed with the Random Dot Stereoacuity Test (Good-Lite Company, Elgin, IL).

*Visual field sensitivity*. Monocular visual fields (left and right eye) were assessed using the Humphrey Field Analyzer (Humphrey Field Analyzer; model HFA-II 750; Carl Zeiss Meditec, Dublin, CA) and the monocular 24–2 Swedish Interactive Threshold Algorithm-Standard. Mean deviation (MD) values were obtained from the tests deemed reliable (i.e., with less than 20% fixation losses and less than 33% false positive and false negative responses). Stage 0 to 1 glaucoma corresponds to no visual field defects (i.e., MD values of 0dB or better) to MD of -6dB.

#### Structural measures

Average cup-to-disc ratio, vertical cup-to-disc ratio, and RNFL thickness measurements for each eye were obtained with spectral domain optical coherence tomography (OCT, model Cirrus; Carl Zeiss Meditec, Dublin, CA), using a 200 x 200 optic disc cube protocol scan.

#### Psychophysical measures

**Fig 2:**
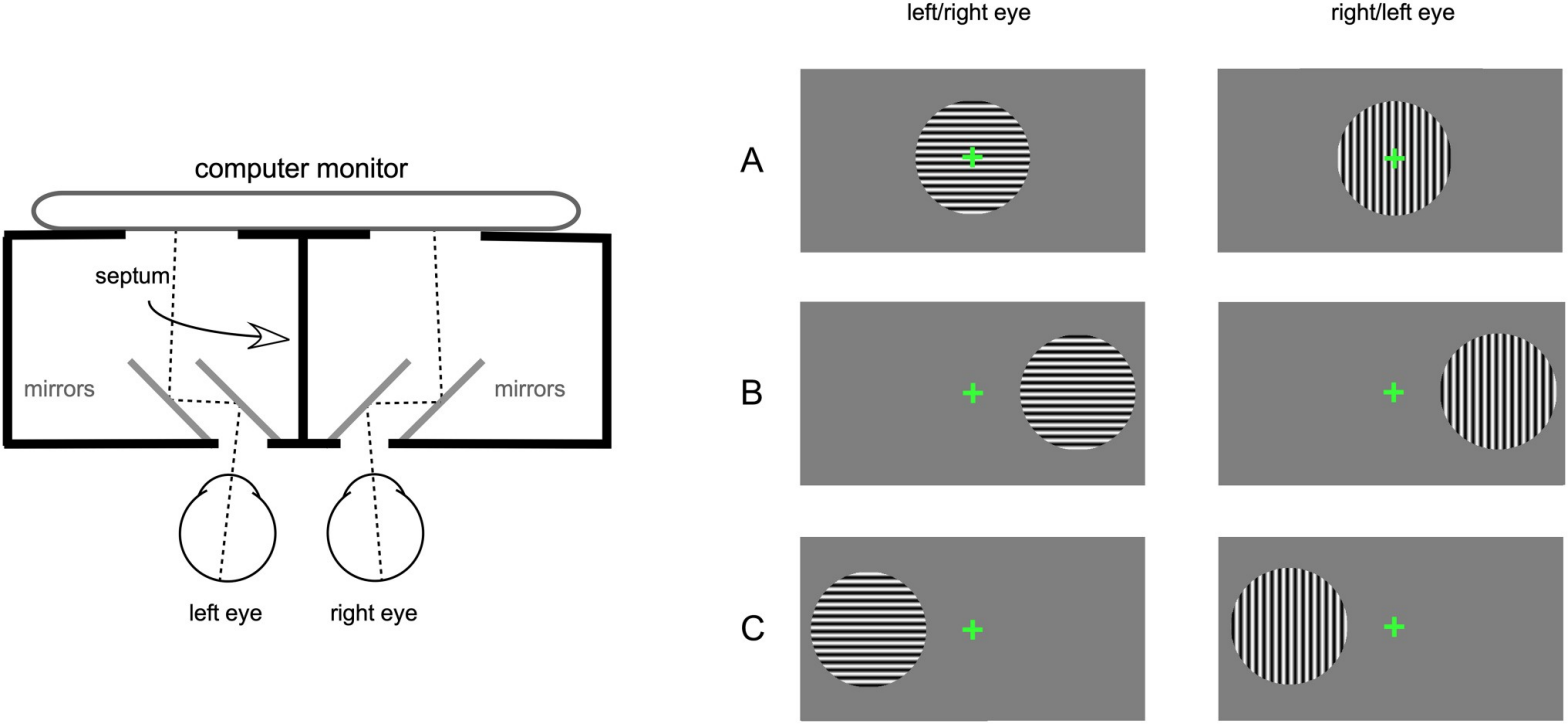
Apparatus and stimuli for the rivalry experiment. Left panel shows a diagram of the double-mirror stereoscope showing the two sets of mirrors (in grey) and the septum which ensured dichoptic presentation of the stimuli. Right panel shows the three types of dichoptic stimuli: A) central, B) 5 deg on the right of the visual field, and C) 5 deg on the left of the visual field. Note that for half of the trials the horizontal stimuli were presented to the left eye and the vertical stimuli to the right eye, and conversely for the other half.

Rivalry rates and percept dominance were obtained during a rivalry test. The rivalry stimuli were generated with VPixx, a graphics and psychophysics software (VPixx Technologies, Inc., Montreal, QC), and presented on an iMac computer screen with a resolution of 1680 x 1050 pixels. A double-mirror stereoscope placed in front of the computer on an adjustable table was used for the dichoptic presentation. A black mask surrounded the stimulus area on the monitor and a vertical septum ensured that different stimuli were seen by the left and the right eyes separately (Fig 2, left panel). The 5 deg diameter stimuli contained orthogonal (horizontal or vertical) sinewave gratings with a spatial frequency of 3 cpd. Stimuli were presented on a gray background either: A) centrally, B) 5 deg eccentrically on the right of the visual field, or C) 5 deg eccentrically on the left (Fig 2, right panel). A 0.5 deg green fixation cross was presented centrally; eccentricity was measured from the middle of this fixation cross to the stimulus’ center.

A button-response box connected to a PC computer and in-house software written in Visual Basic (Microsoft, Albuquerque, NM, US) recorded rivalry rate (i.e., the number of perceptual switches per minute) and percept dominance (i.e., how long each one of the two stimuli or piecemeal percepts were reported). Horizontal and vertical tactile cues were attached to the two response buttons to indicate which button corresponded to each stimulus orientation. Pressing the two simultaneously indicated a piecemeal percept.

### Procedure

Functional, structural, and psychophysical measures as well as the cognitive test were taken in a single 2-hour long session. After the experiment was explained and informed consent was obtained, the participants underwent the following testing: monocular and binocular visual acuity at high and low contrast, stereo acuity thresholds, OCT scans, visual field tests, and cognitive assessment. Then, the binocular rivalry test was conducted as follows. Participants were seated in an adjustable chair and had their head stabilized with a chin rest. The apparatus’ table was adjusted such that the center of the screen was at each participant’s eye level. After all the adjustments were made, a practice run and then testing were performed in a darkened room, where all light sources—except for the computer monitor showing the stimuli—were eliminated. A practice trial used the orthogonal stimuli presented dichoptically in a central location. After it was confirmed that the participants saw only one fixation cross, the participants were instructed to keep their gaze stable on the green cross and asked to indicate whether they perceived the horizontal gratings, the vertical gratings, or a mixed percept by pressing and holding one button, the other button, or both buttons of the button-response box, respectively. Binocular rivalry was then tested in 6 conditions: 3 locations (central, right, left) x 2 stimulus presentation (horizontal gratings to the right eye and vertical gratings to the left eye, and vice versa), shown in a random order. Each condition was one-minute long with a brief break before another condition started. For the peripheral conditions, the participants were repeatedly reminded to keep their eyes fixated on the central green cross, but to pay attention to the eccentric rivalrous stimuli.

### Data analysis

The main outcome measures were the rivalry rate and percept dominance. Rivalry rate was reported as the number of perceptual switches per minute, averaged for the two stimulus presentation conditions (i.e., horizontal gratings presented to the right eye and vertical gratings to the left eye, and vice versa) at each location. The percept dominance was eye-based and defined as the proportion of time of exclusive dominance of the stimulus projected to the left eye, to the right eye, or the mixed percept [i.e., dominant percept / (mixed percept + dominant percepts)]. As with the rivalry rate, the data from the two stimulus presentation conditions were averaged for each location.

Data were analyzed with parametric tests such as Pearson product moment correlations, independent samples t-tests, paired samples t-tests, as one-way analysis of variance (ANOVA) and mixed factorial ANOVA. Alpha level was set at 0.05 for all tests, and, in cases of multiple comparisons, the familywise error rate was controlled with the Bonferroni approach. The ANOVA effects were adjusted with a Greenhouse-Geisser correction when violations of sphericity assumption were detected.

## Results

### Participants

For the two groups, there were no significant differences in age, stereo acuity, visual field’s mean deviation, and MoCA cognitive test (smallest p = 0.08). Binocular and monocular visual acuities at high and low contrast were normal for both groups. At high contrast the average visual acuity was 0.00 ± 0.10 logMAR (Snellen 20/20) or better for the glaucoma group and -0.10 ± 0.11 logMAR (Snellen 20/16) or better for the control group. At low contrast the average visual acuity was 0.12 ± 0.14 logMAR or better for the glaucoma group and 0.03 ± 0.12 logMAR or better for the control group. A large difference between the acuity values at high and low contrast would indicate problems with contrast sensitivity that would not be detectable by visual acuity.[23] The two groups did not differ in this aspect for binocular or monocular viewing (smallest p = 0.52). As expected, the glaucoma group differed significantly from the control group in structural measures: thinner RNFL layer, larger average cup-to-disc ratio, and larger vertical cup-to-disc ratio (smallest t(59) = -3.5, p = 0.001).

#### Inter-ocular differences

In order to ensure that the rivalry results were not due to differences between the two eyes, the inter-ocular differences in functional and structural measures were examined with paired sample t-tests for each group. For the glaucoma group, there were no significant differences between the left and the right eye for acuity at high or low contrast, for visual field’s mean deviation values, for RNFL thickness, for the average cup-to-disc ratio, or for the vertical cup-to-disc ratio (smallest p = 0.2). The results were similar for the control group (smallest p = 0.06). Moreover, the two groups did not differ in any inter-ocular difference measures (smallest p = 0.07).

### Overall analysis of the rivalry rate

**Fig 3:**
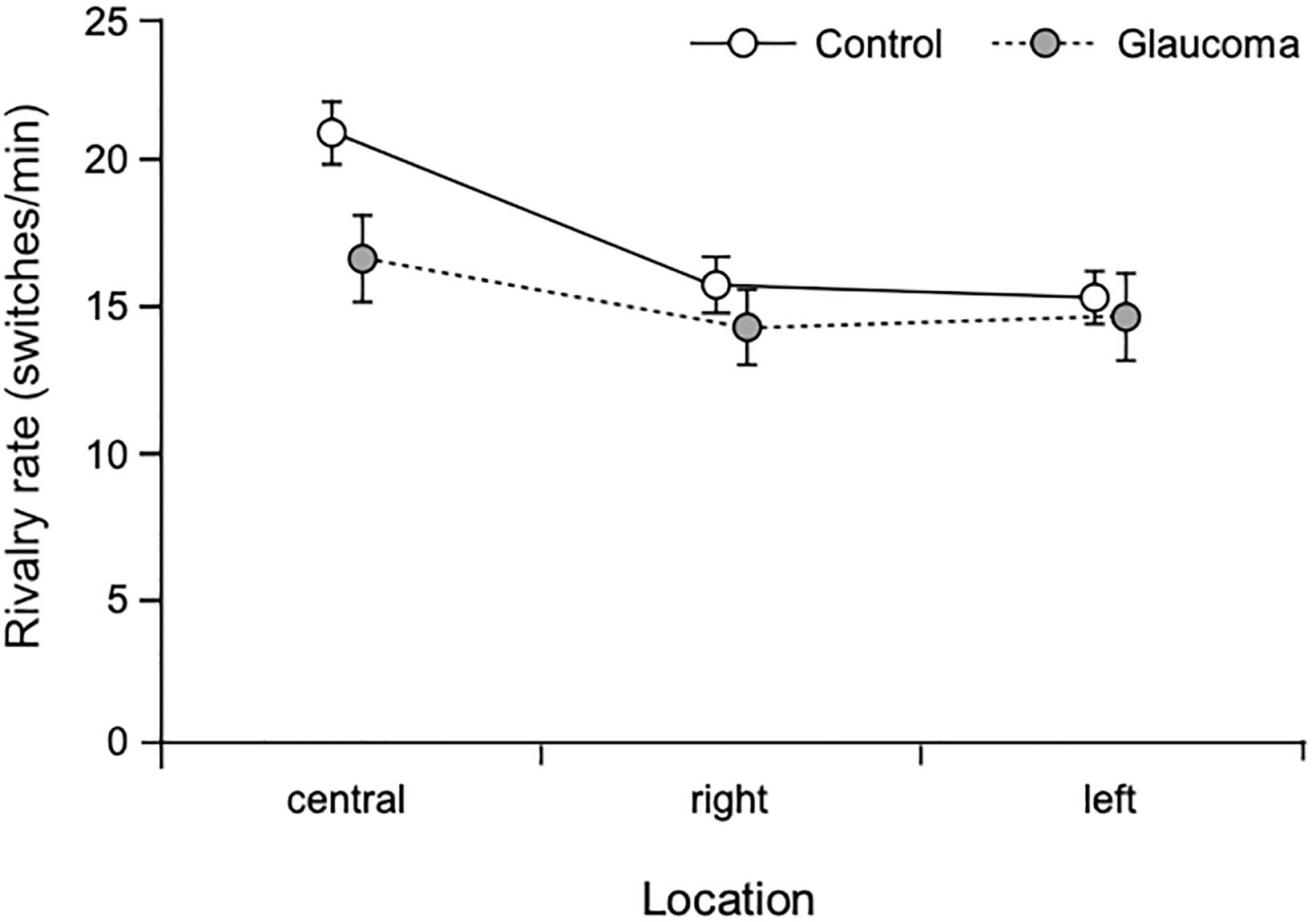
Rivalry rate. Rivalry rate for central and peripheral conditions for the two groups. Error bars are ± 1SE.

Rivalry rate was analyzed with a 3 (Location: central, right, left) x 2 (Group: glaucoma, control) mixed factorial ANOVA. The analysis showed a significant Location main effect F(1.5, 89.8) = 22.5, p < 0.001, partial η^2^ = 0.28 and an interaction Location x Group effect F(1.5, 89.8) = 3.8, p = 0.04, partial η^2^ = 0.06, but no Group effect. Pairwise comparisons showed that overall rivalry rate for the central location was significantly higher than those for peripheral right or left, p < 0.001. Also, rivalry rate of the control group was significantly higher than that of the glaucoma group for the central (p = 0.03), but not for the peripheral locations (smallest p = 0.4). The rivalry rate of the glaucoma group was 20% lower than that of the control group for the central location. For the control group, the rivalry rate was significantly higher in the central condition than in the 2 peripheral conditions, p < 0.001. For the glaucoma group, the rivalry rate was similar for the 3 location conditions, smallest p = 0.05. The results are shown in Fig 3.

### Rivalry rate: NTG vs POAG

**Fig 4:**
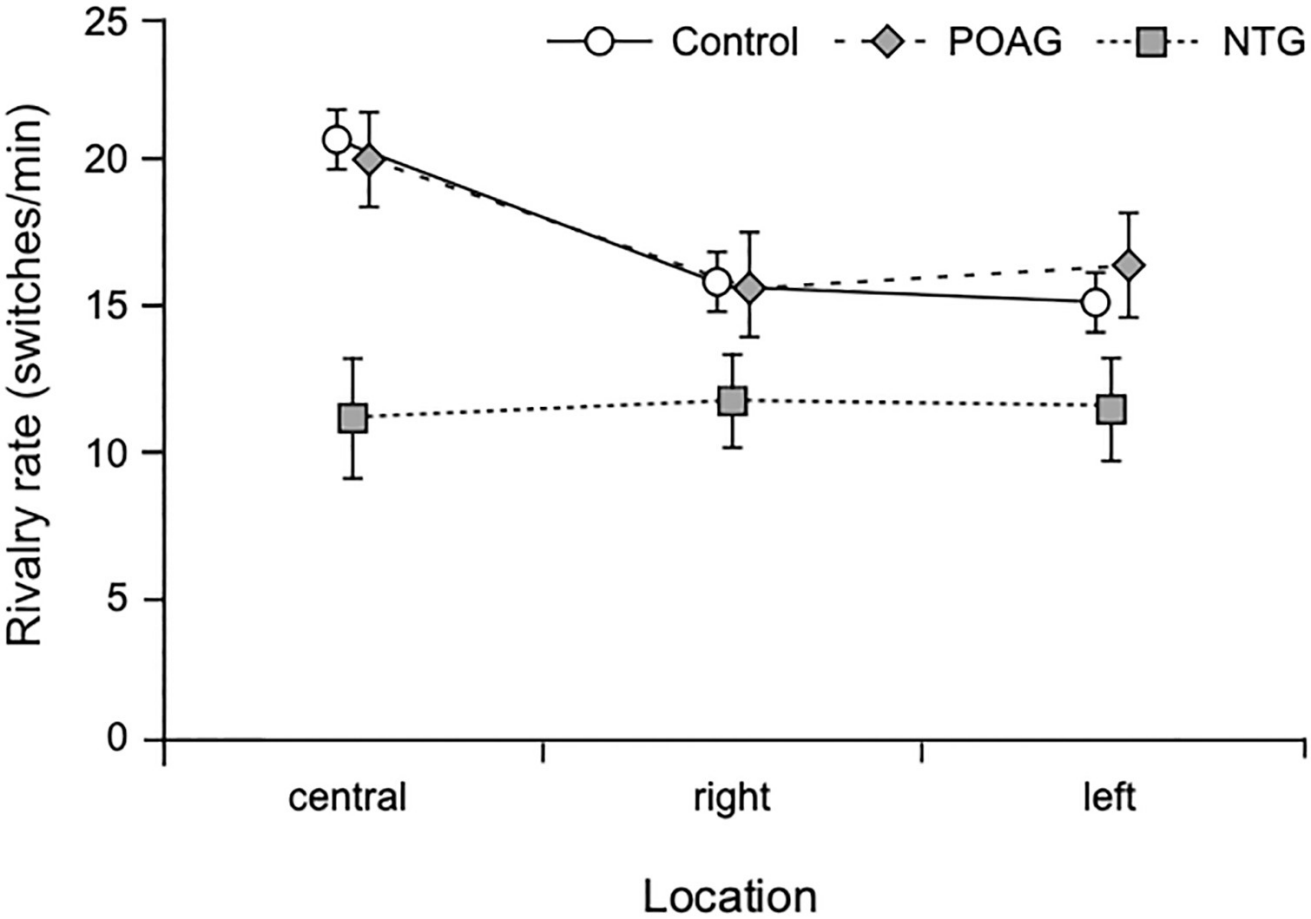
Rivalry rate for subgroups. Rivalry rate for central and peripheral conditions for the control group and the two glaucoma subgroups. Error bars are ± 1SE.

In the glaucoma group, 20 patients had a diagnosis of POAG and 11 patients were diagnosed with NTG; therefore, we split the overall group into 2 subgroups. [The POAG, NTG, and control groups were matched in age, one-way ANOVA F(2, 58) = 1.8, p = 0.17.] We re-analyzed the rivalry rate data using a 3 (Location: central, right, left) x 3 (Group: POAG, NTG, control) mixed factorial ANOVA. The analysis showed a significant main effect of Location, F(1.55, 89.8) = 11.6, p < 0.001, partial η^2^ = 0.17, a significant Location x Group interaction, F (3.1,89.8) = 3.51, p = 0.017, partial η^2^ = 0.11, and a significant Group effect, F(2, 58) = 4.61, p = 0.014, partial η^2^ = 0.14. Follow-up analysis revealed that, overall, rivalry rate was significantly lower for the NTG group than for the POAG (p = 0.017) and control groups (p = 0.028), but the POAG and the control groups had similar rivalry rates. Moreover, as with the previous analysis, overall rivalry rate when the stimuli were in the central location was significantly higher than when they were in the periphery to the left or to the right (largest p = 0.002). The rivalry rate of the NTG group was significantly lower than those of the POAG, p = 0.003 (i.e., 43% lower) and control groups, p < 0.001 (i.e., 46% lower) for the central location, but not for the peripheral locations (smallest p = 0.17). For the NTG group, rivalry rates were similar in all 3 location conditions, whereas POAG and control groups had higher rivalry rates for the central than for the 2 peripheral locations (largest p = 0.028). The results are shown in Fig 4.

### Percept dominance

**Fig 5:**
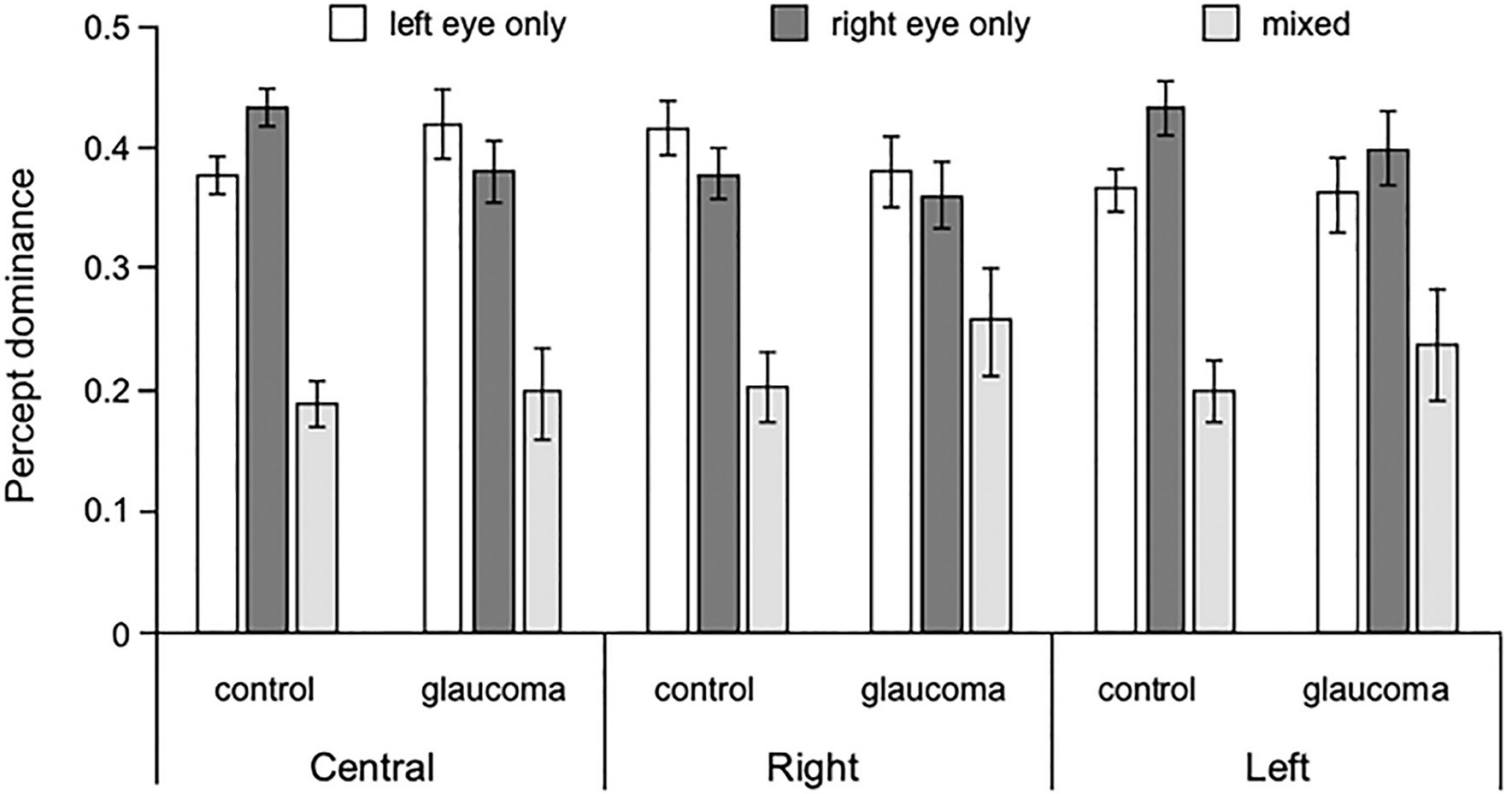
Percept dominance. For the two groups and for stimuli presented centrally, to the right, and to the left, proportion of time of exclusive percept dominance of the stimuli presented to the left and to the right eyes as well as mixed dominance. Error bars are ± 1SE.

The percept dominance was the proportion of time of exclusive dominance of the stimuli projected to the left eye, to the right eye, or the mixed percept. These data were treated as parametric and were analyzed with separate 3 (Percept: left, right, mixed) x 2 (Group: glaucoma, control) mixed factorial ANOVAs for each stimuli location. For the central location, there was only a significant main effect of Percept, F(1.7, 98.4) = 28.5, p < 0.001, partial η^2^ = 0.33, but no interaction or Group effect. Pairwise comparisons showed that the exclusive dominance of the stimuli projected to the right eye was the same as those projected to the left eye, but the mixed percept was perceived significantly less, p < 0.001. The same pattern of results was found for the right location, F(1.4, 85.2) = 11.7, p < 0.001, partial η^2^ = 0.12 and for the left location, F(1.6, 93.5) = 14.6, p < 0.001, partial η^2^ = 0.20. These data are shown in Fig 5.

When the glaucoma group was split into POAG and NTG subgroups the pattern of results was maintained: for the 3 stimuli locations, the separate 3 (Percept: left, right, mixed) x 3(Group: POAG, NTG, control) mixed factorial ANOVAs showed no interaction or Group effect, but a significant Percept effect (largest p = 0.003), with the mixed percept being perceived significantly less (largest p = 0.02) than the exclusive dominance of the stimuli projected to the left or to the right eye.

### Stereo-acuity analysis

The neural mechanisms of binocular rivalry and stereopsis partially overlap.[24] We applied a log transformation to normalize the stereo-acuity data. A one-way ANOVA revealed that the differences in stereo-acuity among the Control, POAG, and NTG groups were not statistically significant, F(2, 58) = 0.99, p = 0.38. We further checked this analysis with a non-parametric test for 3 independent samples using Kruskal-Wallis H test on un-transformed data and found no differences, H(2) = 0.66, p = 0.72.

### Rivalry rate—Central location

Because glaucoma and control groups differed significantly in rivalry rate for the central location, we further examined 1) the relationships of the rivalry rate for this location with functional and structural measures for the two groups, and 2) the ability of the test to detect NTG from overall glaucoma and control groups.

#### Rivalry rate and its relationships with functional and structural measures

For the control group, there was a significant correlation between rivalry rate for the central location and age, r(28) = -0.54, p = 0.002, but this was not the case for the glaucoma group, r(29) = -0.23, p = 0.21. The relationship between rivalry rate and the visual field’s mean deviation (that is, average mean deviations of the left and right eye) was weak but significant for the glaucoma group r(29) = 0.37, p = 0.043, and non-significant for the control group r(28) = -0.25, p = 0.18. There was no significant relationship between rivalry rate and the other functional (i.e., visual acuity, stereo-acuity) or structural (i.e., RNFL thickness, average cup-to-disc ratio, vertical cup-to-disc ratio) measures for both groups (smallest p = 0.054).

#### Ability of the test to detect NTG

**Fig 6:**
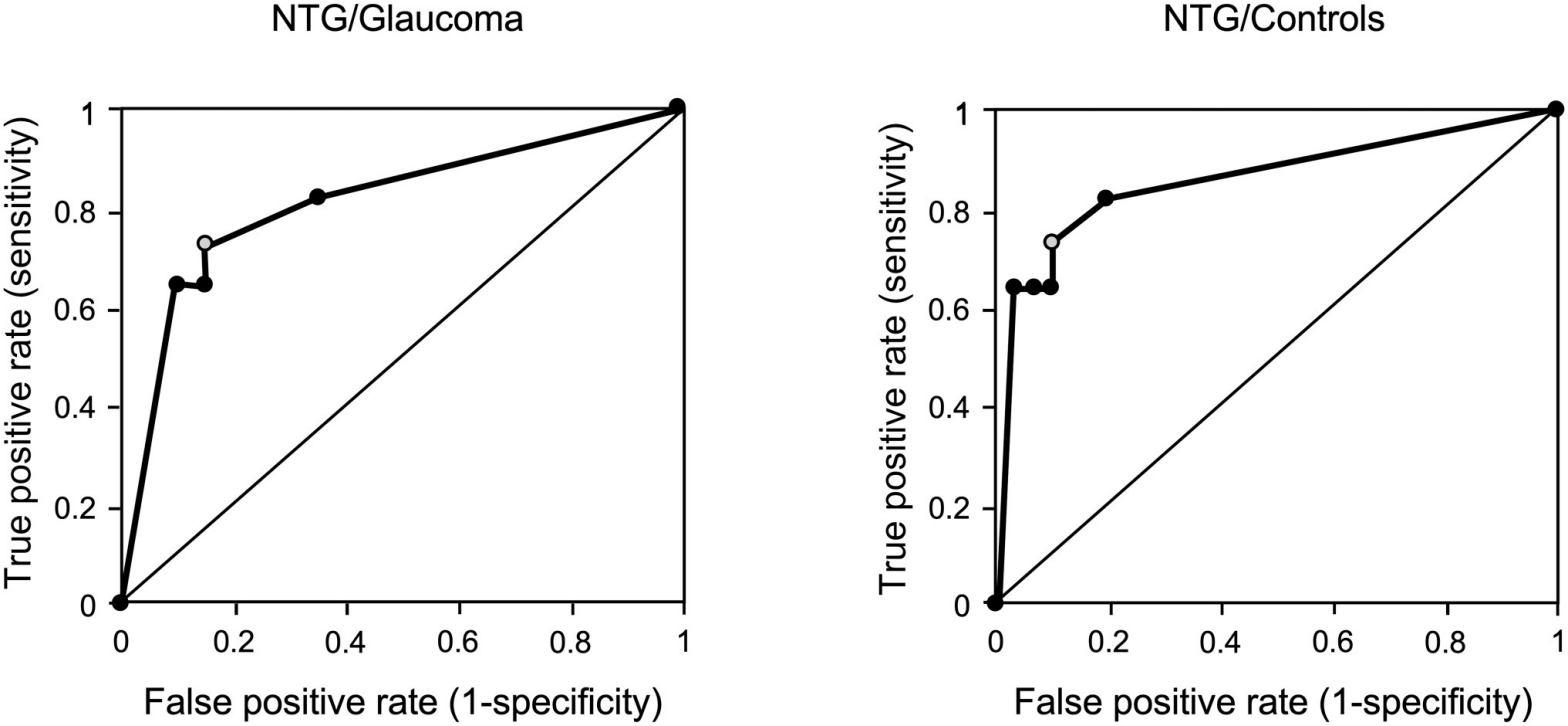
ROC curves. ROC curves are shown for the NTG / Glaucoma model (left panel) and for the NTG / Control model (right panel). The gray marker on both curves represents the best cut-off value.

Rivalry rate for the central location was significantly lower for the overall glaucoma group, but this result was driven by patients with NTG. We further examined how good the test was at separating the patients with NTG from the overall glaucoma group, as well as controls. We first built the receiver operating characteristic (ROC) curves for both models (NTG / Glaucoma and NTG / Control). Then, we determined the best cut-off value based on the Youden’s index, that is the highest value of Sensitivity + Specificity– 1. The ROC curves are shown in Fig 6. We found that for both models, the best cut-off was a value of 14.25. In the NTG subgroup, 8 out of 11 patients had a rivalry rate below this value, whereas only 3 out of 20 patients with POAG and 3 out of 30 controls had the rivalry rate below the cut-off value. Sensitivity, specificity, and accuracy of the two models are shown in Table 2.

**Table 2:** Sensitivity, specificity, and accuracy of the test for detecting NTG from the overall glaucoma group as well as from the control group.

	Sensitivity (%)	Specificity (%)	Accuracy (%)
NTG / Glaucoma	73	85	81
NTG / Control	73	90	85

## Discussion

Using binocular rivalry, we explored the intra- and inter- hemispheric processing of visual information in patients with mild glaucoma who had no significant defects on standard functional measures. Traditional rivalry stimuli (i.e., static orthogonal sinewave gratings) were presented dichoptically in a central location to involve inter-hemispheric processing, and in a peripheral location to the left or to the right to involve processing only in the right or in the left hemisphere, respectively. We found that, compared to the control group, the glaucoma group had a significantly lower rivalry rate for the central location—driven primarily by the patients with NTG—, but the rivalry rates for the peripheral locations were similar for all participants. These results suggest dysfunction in the inter-hemispheric processing of visual information.

It is generally accepted that perceptual dominance dynamics during rivalry involve reciprocal inhibitory interactions between populations of neurons in the visual cortex as well as neural adaptation of the activity associated with the dominant stimulus at any given time. That is, the neural responses associated with the dominant stimulus fade over time, allowing the neural activity associated with the suppressed stimulus to reverse the perceptual dominance.[15,20] The dynamics in the visual cortex during rivalry may depend on a balance of excitatory (i.e., glutamate) and inhibitory (i.e., gamma-aminobutyric acid or GABA) neurotransmitters. It has been shown that in a neurotypical population there is a link between glutamate and GABA levels and rivalry rate,[25] and that higher GABA concentration in the visual cortex leads to a lower rivalry rates.[26] These findings imply that a glutamate and/or a GABA dysregulation in the visual cortex would lead to an abnormal rivalry dynamics; this proposition is supported by research showing lower rivalry rate in neurological disorders associated with GABAergic transmission abnormalities such as bipolar disorder,[27,28] schizophrenia,[29] major depression,[30] attention deficit and hyperactivity disorder,[31] and autism spectrum disorder.[25,32] Glutamate excitotoxicity has been implicated in neurodegenerative diseases such as multiple sclerosis,[33] Alzheimer’s disease, and Huntington’s disease,[34] but binocular rivalry has not been studied in patients affected by these conditions.

Our current results point to a dysfunction of the inter-hemispheric processing involving the corpus callosum, rather than a dysregulation of the excitatory/inhibitory neurotransmitters in the visual cortex. Although GABA dysregulation and glutamate excitotoxicity has been implicated in RGCs degeneration in rodent models of glaucoma,[35–38] and a trend towards higher glutamate levels in the visual cortex with disease progression has been found in humans,[39] the results presented here are not sufficient to support the hypothesis that a neurotransmitter dysregulation that can be detected behaviourally exists in the visual cortex of patients with mild glaucoma. If this were true, then rivalry rate would not be affected selectively for the central location condition (i.e., inter-hemispheric processing), but would also be lower for the peripheral locations (i.e., intra-hemispheric processing). However, in this experiment we measured perceptual switches and percept dominance, but other aspects of binocular rivalry such as speed of propagation of rivalry dominance can be evaluated using different experimental paradigms [40] that may provide further insights into the neural processing during rivalry in glaucoma.

Interestingly, the lower rivalry rate for the central location was driven by patients with NTG. Degeneration in the splenium and body of the corpus callosum has been observed in patients with more advanced stages of NTG,[9] but it is not clear whether the same kind of degeneration exists in POAG. Although metabolic [39] and structural [10] changes have been observed in the glaucomatous brain, it is possible that some white matter changes are specific only to NTG in the initial stages, suggesting a mechanism of disease that may be different from that of POAG.[41] Support for this idea comes from a study showing that, using the diffusion tensor imaging technique, NTG could be discriminated with high accuracy from POAG and controls based on structural changes in the optic radiations.[42] Our study shows that a behavioural test involving inter-hemispheric transfer of visual information is also able to differentiate NTG in a glaucoma group and from controls with acceptable accuracy and with high specificity.

Overall, rivalry rates for peripheral locations were lower than that for the central location. It has been shown that in experienced observers with normal vision the exclusive dominance increases—thus the rivalry rate decreases—with retinal eccentricity. This effect is not explained solely by Troxler fading, spatial frequency, or reduced visual acuity in the periphery, but may be due to a growth in suppression with retinal eccentricity.[43] Our study shows nearly identical peripheral rivalry rates within and between groups and these findings bring additional support to the idea that glaucoma affects inter-hemispheric transfer before any other functional changes.

It has been shown that stereo-acuity starts to decline in glaucoma suspects, [44] but the deficit becomes more pronounced in the later stages of disease.[45] In young observers with healthy binocular vision there is a significant but weak relationship between stereopsis and rivalry rate suggesting that stereopsis and binocular rivalry may share, in a small part, the same neural mechanism.[24] We did not find any relationship between stereo-acuity and rivalry rates for glaucoma or control groups. However, fusion and stereopsis are the main functions of binocular vision and typically they take precedence over binocular rivalry in participants with normal vision [24,46] and perhaps these functions are robust to initial degenerative stages in patients.

In conclusion, there is strong evidence of neurodegeneration in the visual system of patients with early and more advanced stages of glaucoma,[3–10] and recently neurodegeneration has been discovered beyond the primary visual pathways, in the corpus callosum that is involved in inter-hemispheric transfer.[9,10] Using behavioural methods, we probed the efficacy of the inter-hemispheric transfer of visual information and found dysfunction in patients with glaucoma who otherwise had no functional deficits on standard measures. Interestingly, we found that the lower rivalry rate for the central location (i.e., inter-hemispheric processing) was driven by the NTG subgroup, supporting the notion that NTG and POAG pathology do not overlap entirely.

## Supporting information

S1 File Binocular rivalry data for control and glaucoma group. (XLSX)

S2 File Dominance wave propagation during binocular rivalry in mild glaucoma. (DOCX)

## References

[1] Quigley HA, Broman AT. The number of people with glaucoma worldwide in 2010 and 2020. Br J Ophthalmol 2006;90:262–67. 10.1136/bjo.2005.081224 16488940 PMC1856963

[2] Quigley HA. Neural death in glaucoma. Prog Retin Eye Res 1999;18:39–57. 10.1016/s1350-9462(98)00014-7 9920498

[3] Gupta N, Yücel YH. Glaucoma as a neurodegenerative disease. Curr Opin Ophthalmol 2007;18:110–4. 10.1097/ICU.0b013e3280895aea 17301611

[4] Gupta N, Ang L-C, Noel de Tilly L, Bidaisee L, Yucel YH. Human glaucoma and neural degeneration in intracranial optic nerve, lateral geniculate nucleus, and visual cortex. Br J Ophthalmol 2006;90:674–78. 10.1136/bjo.2005.086769 16464969 PMC1860237

[5] Hernowo AT, Boucard CC, Jansonius NM, Hooymans JM, Cornelissen FW. Automated morphometry of the visual pathway in primary open-angle glaucoma. Invest Ophthalmol Vis Sci 2011;52:2758–66. 10.1167/iovs.10-5682 21398286

[6] Garaci FG, Bolacchi F, Cerulli A, Melis M, Spanò A, Cedrone C, et al Optic nerve and optic radiation neurodegeneration in patients with glaucoma: in vivo analysis with 3-T diffusion-tensor MR imaging. Radiology 2009;252:496–501. 10.1148/radiol.2522081240 19435941

[7] Zhang YQ, Li J, Xu L, Zhang L, Wang ZC, Yang H, et al Anterior visual pathway assessment by magnetic resonance imaging in normal-pressure glaucoma. Acta Ophthalmol 2012;90:e295–302. 10.1111/j.1755-3768.2011.02346.x 22489916

[8] Chen WW, Wang N, Cai S, Fang Z, Yu M, Wu Q, et al Structural brain abnormalities in patients with primary open-angle glaucoma: a study with 3T MR imaging. Invest Ophthalmol Vis Sci 2013;54:545–54. 10.1167/iovs.12-9893 23258150

[9] Boucard CC, Hanekamp S, Ćurčić-Blake B, Ida M, Yoshida M, Cornelissen FW. Neurodegeneration beyond the primary visual pathways in a population with a high incidence of normal-pressure glaucoma. Ophthalmic Physiol Opt 2016;36:344–53. 10.1111/opo.12297 27112227

[10] Williams AL, Lackey J, Wizov SS, Chia TM, Gatla S, Moster ML, et al Evidence for widespread structural brain changes in glaucoma: a preliminary voxel-based MRI study. Invest Ophthalmol Vis Sci. 2013;54:5880–7. 10.1167/iovs.13-11776 23838767

[11] Berlucchi G. Visual interhemispheric communication and callosal connections of the occipital lobes. Cortex 2014;56:1–13. 10.1016/j.cortex.2013.02.001 23489777

[12] Kerrigan-Baumrind LA, Quigley HA, Pease ME, Kerrigan DF, Mitchell RS. Number of ganglion cells in glaucoma eyes compared with threshold visual field tests in the same persons. Invest Ophthalmol Vis Sci 2000;41:741–8. 10711689

[13] Alais D, Blake R. Grouping visual features during binocular rivalry. Vision Res 1999;39:4341–53. 10.1016/s0042-6989(99)00146-7 10789428

[14] Lee SH, Blake R. Rival ideas about binocular rivalry. Vision Res 1999;39:1447–54. 10.1016/s0042-6989(98)00269-7 10343813

[15] Blake R, Wilson HR. Binocular vision. Vision Res 2011;51:754–70. 10.1016/j.visres.2010.10.009 20951722 PMC3050089

[16] Lee SH, Blake R, Heeger DJ. Hierarchy of cortical responses underlying binocular rivalry. Nat Neurosci. 2007;10:1048–54. 10.1038/nn1939 17632508 PMC2615054

[17] Miller SM, Liu GB, Ngo TT, Hooper G, Riek S, Carson RG, et al Interhemispheric switching mediates perceptual rivalry. Curr Biol 2000;10:383–92. 10.1016/s0960-9822(00)00416-4 10753744

[18] Tong F, Meng M, Blake R. Neural bases of binocular rivalry. Trends Cogn Sci 2006;10:502–11. 10.1016/j.tics.2006.09.003 16997612

[19] O’Shea RP, Corballis PM. Binocular rivalry in split-brain observers. J Vis 2003;3:610–5. 10.1167/3.10.3 14640884

[20] Wilson HR. Computational evidence for a rivalry hierarchy in vision. Proc Natl Acad Sci U S A. 2003;100:14499–503. 10.1073/pnas.2333622100 14612564 PMC283620

[21] O’Shea RP, Corballis PM. Visual grouping on binocular rivalry in a split-brain observer. Vision Res 2005;45:247–61. 10.1016/j.visres.2004.08.009 15581923

[22] Ritchie KL, Bannerman RL, Turk DJ, Sahraie A. Eye rivalry and object rivalry in the intact and split-brain. Vision Res 2013;91:102–7. 10.1016/j.visres.2013.08.004 23973439

[23] Colenbrander A. Vision rehabilitation is part of AMD care. Vision 2018;2(4):1–23. 10.3390/vision2010004 31735868 PMC6835661

[24] Halpern DL, Patterson R, Blake R. Are stereoacuity and binocular rivalry related? Am J Optom Physiol Opt. 1987;64:41–4. 10.1097/00006324-198701000-00007 3826276

[25] Robertson CE, Ratai EM, Kanwisher N. Reduced GABAergic action in the autistic brain. Curr Biol. 2016;26:80–5. 10.1016/j.cub.2015.11.019 26711497

[26] van Loon AM, Knapen T, Scholte HS, St John-Saaltink E, Donner TH, Lamme VA. GABA shapes the dynamics of bistable perception. Curr Biol. 2013;23:823–7. 10.1016/j.cub.2013.03.067 23602476

[27] Pettigrew JD, Miller SM. A ‘sticky’ interhemispheric switch in bipolar disorder? Proc. R. Soc. Lond. B Biol. Sci. 1998;265:2141–8. 10.1098/rspb.1998.0551 PMC1689515 9872002

[28] Miller SM, Gynther BD, Heslop KR, Liu GB, Mitchell PB, Ngo TT, et al Slow binocular rivalry in bipolar disorder. Psychol Med. 2003;33:683–92. 10.1017/s0033291703007475 12785470

[29] Xiao G, He K, Chen X, Wang L, Bai X, Gao L, et al Slow Binocular rivalry as a potential endophenotype of schizophrenia. Front Neurosci. 2018 9 12;12:634 10.3389/fnins.2018.00634 30258349 PMC6143673

[30] Jia T, Ye X, Wei Q, Xie W, Cai C, Mu J, et al Difference in the binocular rivalry rate between depressive episodes and remission. Physiol Behav. 2015;151:272–8. 10.1016/j.physbeh.2015.08.007 26247392

[31] Amador-Campos JA, Aznar-Casanova JA, Ortiz-Guerra JJ, Moreno-Sánchez M, Medina-Peña A. Assessing attention deficit by binocular rivalry. J Atten Disord. 2015;19:1064–73. 10.1177/1087054713482686 23569154

[32] Robertson CE, Kravitz DJ, Freyberg J, Baron-Cohen S, Baker CI. Slower rate of binocular rivalry in autism. J Neurosci. 2013;33:16983–91. 10.1523/JNEUROSCI.0448-13.2013 24155303 PMC3807027

[33] Werner P, Pitt D, Raine CS. Multiple sclerosis: altered glutamate homeostasis in lesions correlates with oligodendrocyte and axonal damage. Ann Neurol. 2001;50:169–80. 10.1002/ana.1077 11506399

[34] Lewerenz J, Maher P. Chronic glutamate toxicity in neurodegenerative diseases-What is the Evidence? Front Neurosci. 2015;9:469 10.3389/fnins.2015.00469 26733784 PMC4679930

[35] Moreno MC, de Zavalía N, Sande P, Jaliffa CO, Fernandez DC, Keller Sarmiento MI, et al Effect of ocular hypertension on retinal GABAergic activity. Neurochem Int. 2008;52:675–82. 10.1016/j.neuint.2007.08.014 17928106

[36] Okumichi H, Mizukami M, Kiuchi Y, Kanamoto T. GABA A receptors are associated with retinal ganglion cell death induced by oxidative stress. Exp Eye Res. 2008;86:727–33. 10.1016/j.exer.2008.01.019 18336815

[37] Gong JL, Lou XT, Yuan YX, Chen LW, Ji PT, Li L, et al The increased expression of GABA receptors within the arcuate nucleus is associated with high intraocular pressure. Mol Vis. 2018;24:574–586. 30174387 PMC6107798

[38] Nguyen D, Alavi MV, Kim KY, Kang T, Scott RT, Noh YH, et al A new vicious cycle involving glutamate excitotoxicity, oxidative stress and mitochondrial dynamics. Cell Death Dis. 2011;2:e240 10.1038/cddis.2011.117 22158479 PMC3252734

[39] Murphy MC, Conner IP, Teng CY, Lawrence JD, Safiullah Z, Wang B, et al Retinal structures and visual cortex activity are impaired prior to clinical vision loss in glaucoma. Sci Rep. 2016;6:31464 10.1038/srep31464 27510406 PMC4980591

[40] Wilson HR, Blake R, Lee SH. Dynamics of travelling waves in visual perception. Nature. 2001;412:907–10. 10.1038/35091066 11528478

[41] Nuzzi R, Dallorto L, Rolle T. Changes of visual pathway and brain connectivity in glaucoma: A systematic review. Front Neurosci. 2018;12:363 10.3389/fnins.2018.00363 29896087 PMC5986964

[42] El-Rafei A, Engelhorn T, Wärntges S, Dörfler A, Hornegger J, Michelson G. Glaucoma classification based on visual pathway analysis using diffusion tensor imaging. Magn Reson Imaging. 2013;31:1081–91. 10.1016/j.mri.2013.01.001 23751976

[43] Blake R, O’Shea RP, Mueller TJ. Spatial zones of binocular rivalry in central and peripheral vision. Vis Neurosci. 1992;8:469–78. 10.1017/s0952523800004971 1586647

[44] Gupta N, Krishnadev N, Hamstra SJ, Yücel YH. Depth perception deficits in glaucoma suspects. Br J Ophthalmol. 2006;90:979–81. 10.1136/bjo.2006.091025 16672326 PMC1857183

[45] Lakshmanan Y, George RJ. Stereoacuity in mild, moderate and severe glaucoma. Ophthalmic Physiol Opt. 2013;33:172–8. 10.1111/opo.12021 23297812

[46] Blake R, Boothroyd K. The precedence of binocular fusion over binocular rivalry. Perception & Psychophysics 1985;37:114–124. 4011364 10.3758/bf03202845

