# Supplementary material for: Intra- and inter-hemispheric processing during binocular rivalry in mild glaucoma
Source: PLoS One. 2020 Feb 25;15(2):e0229168. doi: 10.1371/journal.pone.0229168 (PMC7041812; doi:10.1371/journal.pone.0229168)
Supplement: S2 File — (DOCX) [file pone.0229168.s002.docx]

Dominance wave propagation during binocular rivalry in mild glaucoma

Luminita Tarita-Nistor^1^ PhD, Saba Samet^1,2^ BSc, Graham E Trope^1,3^ MD, PhD,

Esther G González^1,3^ PhD

^1^Krembil Research Institute, Toronto, Canada; ^2^Faculty of Medicine, University of Toronto, Canada; ^3^Ophthalmology and Vision Science, University of Toronto, Canada

Corresponding author:

Luminita Tarita-Nistor, PhD

Krembil Research Institute

Toronto Western Hospital

399 Bathurst St, FP 6-212

Toronto, ON, M5T 2S8

[lumi.tarita-nistor@rogers.com](mailto:lumi.tarita-nistor@rogers.com)

Phone: +1 416-603-5800 x 2515

**Highlights**

Dominance waves of binocular rivalry were examined in mild glaucoma and controls

The efficacy of inter-hemispheric transfer was probed by measuring wave propagation

There was disruption of wave propagation in mild glaucoma

The results point to possible deficits of callosal function in mild glaucoma

**Abstract**

Glaucoma is both a progressive optic neuropathy and a neurodegenerative disease affecting structures in the primary visual pathway. Other vision-associated areas may also be affected, including the corpus callosum which is involved in inter-hemispheric transfer. This study evaluated dominance wave propagation during binocular rivalry to probe transfer efficacy of the corpus callosum in 20 patients with mild open angle glaucoma and 25 age-matched controls. The two groups were matched for functional measures such as stereo-acuity, binocular visual acuity, and visual field mean deviation. Monocular functional and structural measures were equivalent for the left and right eye of each participant. Using Wilson et al.’s travelling wave paradigm [Nature, 412 (2001) 907–910], intra- and inter-hemispheric failure rates of traveling wave transmission and the travelling wave propagation times were recorded for the two groups. For the control group, the wave propagation failure rate was significantly greater for the inter- than for the intra-hemispheric condition (p < 0.001), but for the glaucoma group, the failure rates were equally high for the two conditions. The wave propagation time was significantly longer for the inter- than for the intra-hemispheric condition for the control group (p = 0.014), while the opposite was true for the glaucoma group (p = 0.012). These results imply strong cortical inhibitory action and increased excitability of callosal transfer in patients who otherwise have normal performance on standard functional measures.

**Key words:** glaucoma, travelling wave, binocular rivalry, inter-hemispheric transfer, corpus callosum

**Introduction**

Glaucoma is a progressive, degenerative eye disease and the leading cause of irreversible vision loss in people over 40 years of age (Quigley & Broman, 2006; Varma et al., 2011). Despite preserved good central vision until later stages, patients with glaucoma show impairments in many aspects of visual processing, in ocular motor control, and in functional vision. For example, for lower levels of visual processing these patients exhibit higher motion detection thresholds (Silverman et al, 1990; Bullimore et al., 1993; Trick et al, 1995; Westcott et al., 1998; McKendrick et al., 2005), longer latency for vection responses (Tarita-Nistor et al., 2014; Brin et al., 2019), reduced contrast sensitivity (Hawkins et al., 2003; McKendrick et al., 2007), and decreased stereopsis (Essock et al., 1996). Changes in the ocular motor system are reflected by abnormal saccadic eye-movement control (Kanjee et al., 2012; Lamirel et al., 2014), while functional changes are shown in atypical eye-hand coordination (Kotecha et al., 2009), reduced reading abilities (Ramulu et al., 2009; Ramulu et al., 2013), difficulties navigating the environment (Turano et al., 1999; Friedman et al., 2007; Ramulu, 2009), and increased risk of falls (Black et al., 2011) and motor vehicle collisions (Haymes et al., 2007).

Physiologically, glaucoma is characterized by progressive loss of retinal ganglion cells (RGCs), but the exact mechanism of the disease remains to be elucidated (Quigley, 1999). The RGCs damage is propagated — through the mechanism of Wallerian degeneration — in all the neural structures of the primary visual system, including the retina, optic nerve, optic chiasm, optic tract, lateral geniculate nucleus, optic radiation, and the visual cortex (Gupta et al., 2006; Garaci et al., 2009; Hernowo et al., 2011; Zhang et al., 2012; Chen et al., 2013; Boucard et al., 2016). Recent findings suggest the neurodegeneration is not limited to the primary visual system, but that there are widespread structural changes in the brain, including in the corpus callosum — the largest white matter bundle connecting the two brain hemispheres (Williams et al., 2013; Boucard et al., 2016). In some brain structures neurodegeneration associated with glaucoma depends on disease severity in an unusual way: for example, when compared to controls, the volume of the corpus callosum is significantly larger in early glaucoma but significantly smaller in moderate and advanced stages of the disease. It has been suggested that the increase in the volume of the brain structures in the initial stages of the disease may be indicative of either an inflammatory response to neuronal injury, or — although unlikely — of neuroplasticity (Williams et al., 2013). Nevertheless, these results point to a degenerative mechanism in glaucoma independent from that due to the propagation of RGC damage.

The corpus callosum is the most important structure involved in inter-hemispheric transfer and its efficacy can be probed using the phenomenon of binocular rivalry. When two dissimilar stimuli are presented in retinal correspondence and viewed dichoptically so that each eye sees exclusively one stimulus, the brain cannot combine the two images into a unified percept. Instead, the two stimuli rival for perceptual dominance with only one being perceived briefly and then the dominance switches to the previously suppressed stimulus, in a continuous cycle (Alais & Blake, 1999; Lee & Blake, 1999; Miller et al., 2000; Tong et al., 2006; Lee et al., 2007; Blake & Wilson, 2011). When the two stimuli are projected to only one hemifield, rivalry is processed in the contralateral hemisphere, but when the stimuli are projected to both hemifields binocular rivalry processes in the two hemispheres need to be synchronized by the corpus callosum (O’Shea & Corballis, 2003). Changes in perceptual dominance happens gradually particularly for larger stimuli (Blake et al., 1992); these transition waves of perceptual dominance can be examined using the Wilson et al.’s (2001) travelling wave paradigm (see Methods section for a detailed description of this paradigm). Depending on the experimental setup, binocular rivalry can be used as a tool for investigating the intra- and inter-hemispheric processing of visual information.

In healthy observers, inter-hemispheric travelling wave propagation takes on average 173 ms longer than intra-hemispheric wave propagation, and this perhaps represents the time penalty required to cross the long-range callosal fibers (Wilson et al., 2001). The inter-hemispheric travelling wave is propagated only through the callosal fibers of the splenium that connect the left and right V1 (Genç et al., 2011), suggesting than anomalies of the wave dynamics would be indicative of callosal damage. Indeed, patients with mild traumatic brain injury — who are especially susceptible to long axonal damage —show high failure rate of travelling wave transmission but, counterintuitively, the propagation time is shorter inter- than intra-hemispherically (Spiegel et al., 2015).

The purpose of this study was to probe the transfer efficacy of the corpus callosum in patients with mild glaucoma who otherwise have no functional deficits using Wilson et al.’s (2001) travelling wave paradigm. We hypothesized that the inter-hemispheric travelling wave dynamics are affected in these patients, which would imply callosal dysfunction.

**Materials and methods**

***Participants***

Participants were 20 patients with bilateral mild open angle glaucoma (mean age 65 ± 12 years) recruited from the Eye Clinic at the Toronto Western Hospital, Toronto, Canada, and 25 age-matched controls with healthy vision (mean age 63 ± 10 years) recruited from ads posted around the same hospital, volunteers or staff members. All patients had a confirmed diagnosis given by a glaucoma specialist, were under pharmaceutical treatment to normalize intraocular pressure, and seen on a regular basis to monitor disease progression. Based on their visual field test, the patients’ glaucoma severity was classified as stage 0 to 1 on the Hodapp-Parrish Anderson Glaucoma Grading Scale, which corresponds to no or mild visual field loss. All participants had no other ocular pathologies with the exception of symmetric mild cataract, no significant monocular or binocular functional deficits, and no significant functional or structural asymmetries in the two eyes. All participants with a history of neurological diseases or cognitive impairment were excluded. Fourteen patients had a diagnosis of primary open angle glaucoma (POAG) and 6 had normal tension glaucoma (NTG). The research was approved by the institutional ethics board and conducted in accordance with the tenets of the declaration of Helsinki. Written informed consent was obtained from all participants. Clinical and demographic characteristics of the two groups shown in Table 1 and a detailed analysis of the structural and functional measures are presented in the Results section.

***Apparatus and Stimuli***

***Functional and structural measures***

The following functional measures were obtained: 1) monocular and binocular visual acuity at high (95%) and low contrast (25%) at a distance of 6m with a computerized version of the ETDRS (Early Treatment Diabetic Retinopathy Study) chart (single line) using the Accommodata Stimuli System, Version 3.5 (Haag–Streit, Mason, OH) and a letter-by-letter scoring system; 2) stereo acuity with the Random Dot Stereoacuity Test (Good-Lite Company, Elgin, IL); and 3) monocular visual field sensitivity (mean deviation or MD) for each eye with the Humphrey Field Analyzer (Humphrey Field Analyzer; model HFA-II 750; Carl Zeiss Meditec, Dublin, CA) using the 24-2 Swedish Interactive Threshold Algorithm-Standard. Cognitive function evaluation was performed with the Montreal Cognitive Assessment test (MoCA, www.mocatest.org). Structural measures were obtained for each eye with the spectral domain optical coherence tomography (OCT, model Cirrus; Carl Zeiss Meditec, Dublin, CA) using a 200 x 200 optic disc cube protocol scan, and included average cup-to-disc ratio, vertical cup-to-disc ratio, and peripapillary retinal nerve fiber layer (RNFL) thickness.

***Psychophysical measures***

We used Wilson et al.’s (2001) travelling wave paradigm and our version of the stimuli — shown in Figure 1 — was similar to that used by Genç et al. (2011) and Siegel et al. (2015). In short, one low contrast ring-shaped stimulus and one high contrast stimulus were presented dichoptically using a double mirror stereoscope. The low contrast stimulus was the “target” and presented first. Shortly after, the high contrast stimulus was presented to the other eye. Typically, this suppresses the target stimulus completely. Next, a local increment in contrast (a “trigger”) of the target stimulus was presented to facilitate the propagation of dominance of this stimulus. The trigger had 4 angular locations relative to the arriving point (the two black dots in Figure 1), but the distance between the trigger and the arriving point was always 120 deg in polar coordinates. The trigger and arriving point were either in the same or in different hemifields. While keeping a steady fixation on the bullseye pattern, participants pressed the spacebar when the travelling wave reached the arriving point by the shortest angular distance. If no travelling wave was initiated, participants were instructed to wait for the next trial. The computer program registered the time of travelling wave propagation on the short arc and the travelling wave transmission failure rate. Two versions of the test were created: version A with the arriving point situated at 130 deg (lower visual field) and version B with the arriving point situated at 310 deg (upper visual field). Within each version, there were 4 conditions (2 locations of the trigger x 2 kinds of dichoptic presentations) with 10 replications per condition. This produced a total of 40 trials that were randomly presented. The trigger’s location was chosen to be either in the same hemifield as the arriving point (i.e., intra-hemispheric processing) or in different hemifields (i.e., inter-hemispheric processing). For version A, the trigger was shown either at 10 deg or at 250 deg and for version B the trigger was located either at 190 deg or at 70 deg. One version of the test (i.e., A or B) was chosen randomly for each participant.


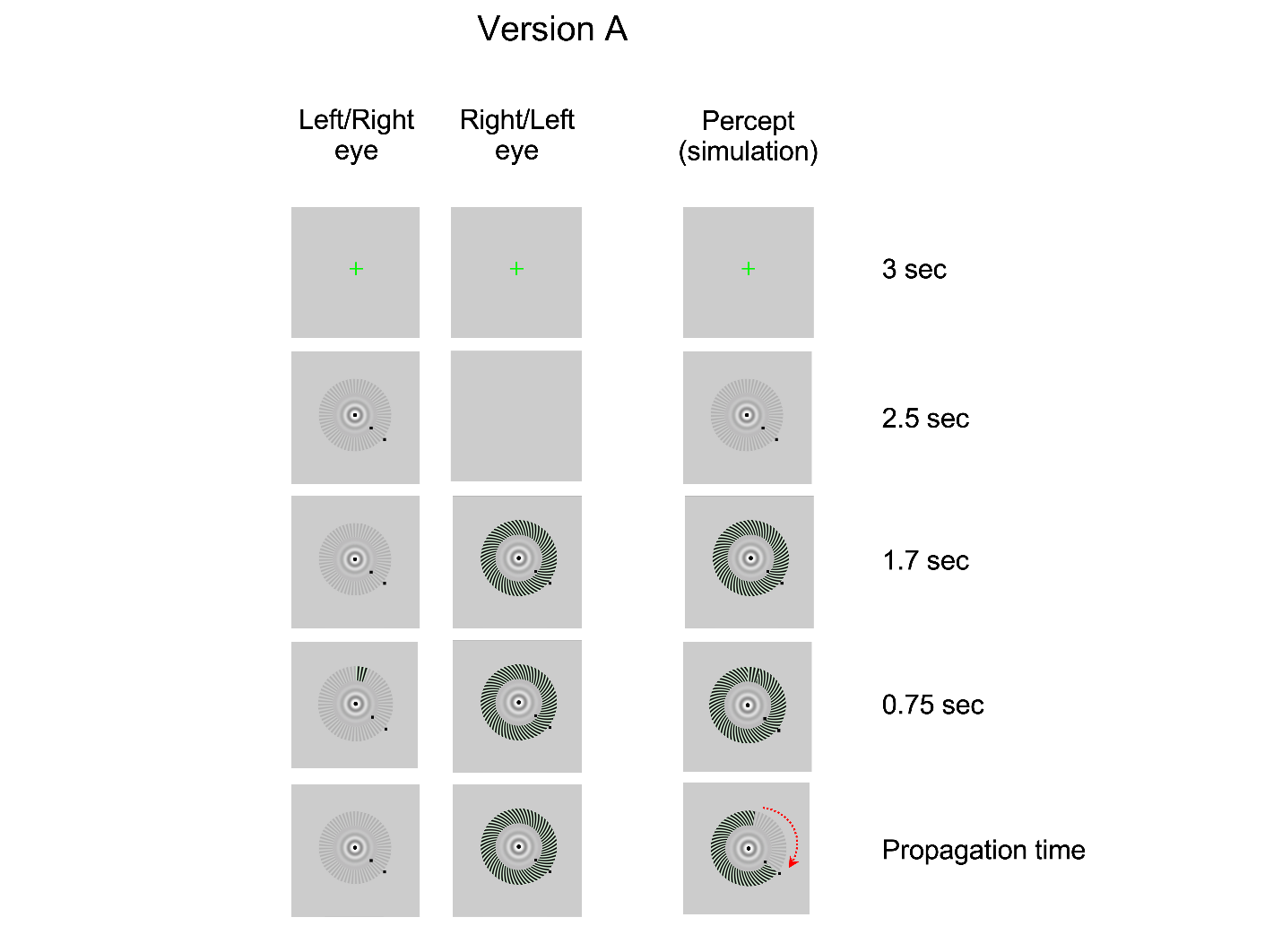


Figure 1. Stimuli for travelling wave initiation, shown dichoptically. This figure shows version A of the experiment. Time sequence of stimulus presentation is shown on the right column.

Figure 1 shows the stimuli. The strokes of the green fixation cross were 1 deg in height and 0.1 deg in width. The central bullseye section was a Gabor patch consisting of an 0.8 cpd concentric sinusoidal grating with a Gaussian envelope with a standard deviation of 1.5 deg. The fixation circle was a black disc 0.7 deg in diameter. The target (i.e., low contrast stimulus) and high contrast stimulus that produced the rivalry had an inner diameter of 4 deg and an outer diameter of 6.5 deg. They both included radial spiral bars with a sinusoidal profile and a spatial frequency of 60 cpd. The high contrast stimulus was green and had an additional spiral component. The trigger had an angular length of 19 deg, was high contrast, green, and had no spiral component. VPixx, a graphics and psychophysics software (http://www.vpixx.com), was used for stimulus design, controlling the experiment, and recording the data.

***Procedure***

All participants were tested during a single 2-hour session as follows. First, the study was explained in detail and informed consent obtained. Second, functional (i.e., monocular and binocular visual acuity at high and low contrast, stereo acuity thresholds, and monocular visual field sensitivity for each eye), structural (i.e., RNFL layer, average cup-to-disc ratio, vertical cup-to-disc ratio for each eye), and cognitive (i.e., MoCA test) measures were obtained. Finally, the psychophysical test was conducted in a darkened room with the computer screen as the only light source. For this, participants had their head stabilized with a chin rest and the apparatus positioned such that the centre of the screen was at eye level using an adjustable table. It was verified that the participants were able to fuse two fixation crosses presented dichoptically. The experiment was explained using a step-by-step visual demonstration of the stimuli and the possible visual percepts. This demonstration was presented as many times as the participants needed to understand the task. Then, the 40 trial experiment — version A or B, randomly selected — began.

Each trial began with a 3s fixation period, after which the low contrast stimulus was presented to one eye and then the high contrast stimulus to the other eye after a 2.5s delay. After 1.7s from the high contrast stimulus presentation, the trigger appeared on the target (i.e., the low contrast stimulus) for 0.75s. Once the trigger disappeared, the participants were instructed to press the spacebar of a keyboard if and only if they saw the travelling wave reaching the arriving point on the short arc; if the travelling wave was initiated but did not reach the arriving point, or the wave went on the long arc, or there was no travelling wave initiated, they were instructed not to press the spacebar and to wait for the next trial. Each trial was 20s long in total, with a time to failure of 12.04s. The experiment proceeded with no breaks, but was restarted any time a participant felt he or she did not understand the task. Figure 2 shows a schematic of the trials.


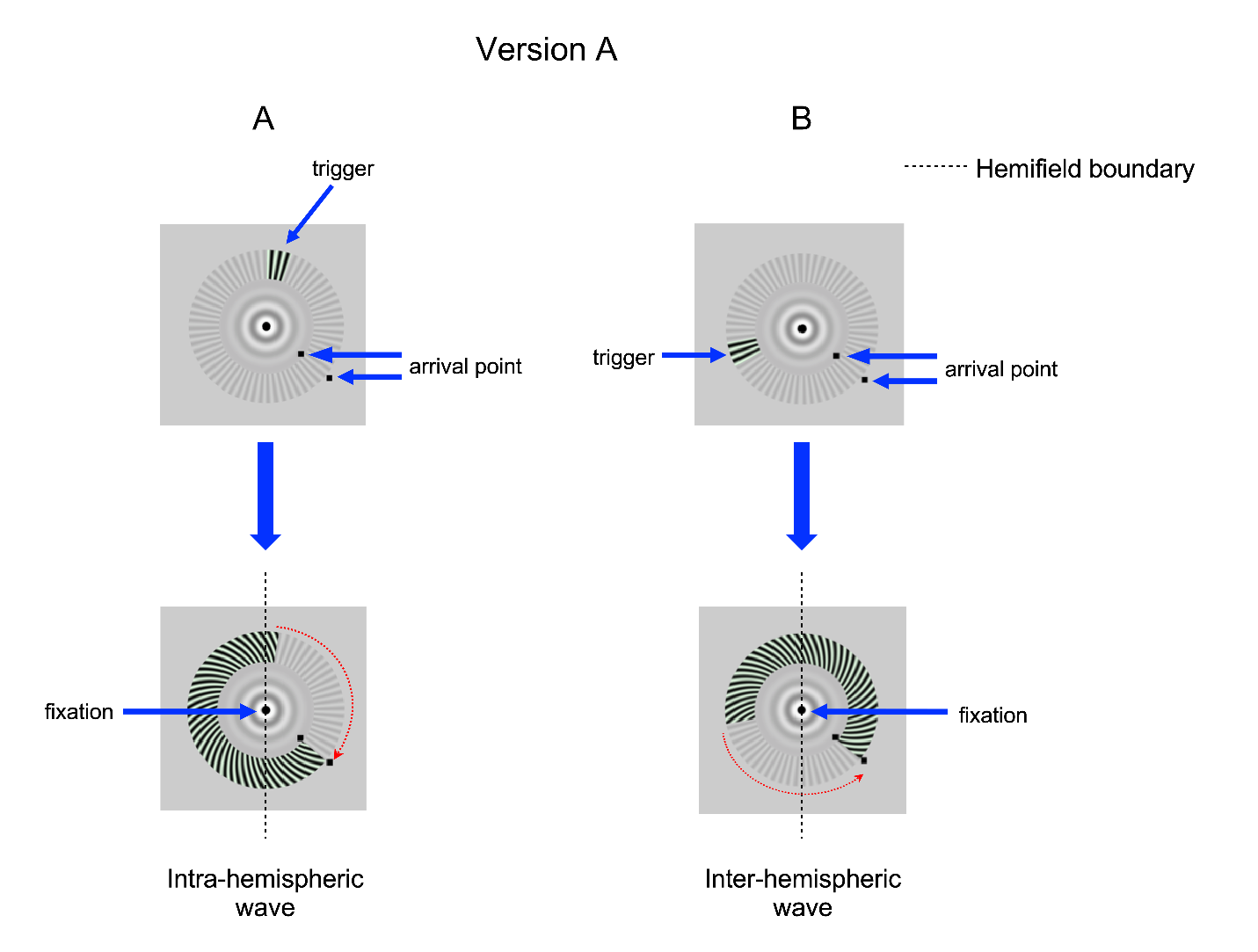


Figure 2. Schematics of the travelling wave propagation for intra-hemispheric (left panel) and inter-hemispheric (right panel) condition, shown on version A of the experiment. The top figures show the trigger presented on the target stimulus. The bottom figures show the successful wave propagation along the short arc to the arriving point.

***Data analysis***

The main outcome measures were 1) travelling wave transmission failure rate and 2) time of travelling wave propagation, for both intra- and inter-hemispheric conditions. An Intra/Inter Ratio — defined as the ratio of the intra-/inter-hemispheric performance — was also computed. Data were analyzed primarily with parametric tests such as independent samples t-tests, paired samples t-tests, and mixed factorial analyses of variance (ANOVAs). When the sphericity assumption was violated, the ANOVA effects were adjusted with a Greenhouse-Geisser correction. The familywise error rate was controlled with the Bonferroni approach when multiple comparisons were performed. In isolated instances of high variability in the data, the Mann-Whitney U non-parametric test was used. An alpha level of 0.05 was used for all tests.

**Results**

***Participants: Functional and structural differences***

Because the rivalry results could be affected by asymmetries between the right and the left eye, we assessed the functional and structural measures within each group with paired-samples t-tests. Functional measures of each eye included visual fields mean deviation, and visual acuity at high and low contrast. Structural measures included RNFL measurements, average cup-to-disc ratio, and vertical cup-to-disc ratio. The left eye was not significantly different from the right eye on any of the functional and structural measures, for the glaucoma group (smallest p = 0.08) and for the control group (smallest p = 0.06).

We further examined the binocular functional differences between the two groups with independent samples t-tests. There were no significant differences in stereo-acuity (p = 0.50) or binocular acuity at high (p = 0.051) and low (p = 0.09) contrast. Monocular functional differences between the two groups were also examined. The visual field’s mean deviation of the left eye as well as that of the right eye were not significantly different between the two groups (smallest p = 0.14). Monocular visual acuities were normal in both groups, with averages slightly better than 0.0 logMAR (Snellen 20/20) at high contrast. The differences between groups in monocular acuity were in general statistically (p value range 0.02 to 0.08) — but never clinically — significant: the largest difference was of 0.08 logMAR (or 4 letters) for the right eye’s visual acuity at low contrast. The functional and structural measures of the two groups are shown in Table 1.

Table 1. Demographic and clinical characteristics of the glaucoma and control group.

|  | Glaucoma | Control | p value |
| --- | --- | --- | --- |
| N [M/F] | 20 [12/8] | 25 [16/9] | - |
| Age (years) | 65 ± 12 | 63 ± 10 | 0.50 |
| Stereo acuity (sec) | 40 ± 34 | 29 ± 30 | 0.24 |
| Visual acuity 96% contrast (logMAR) | |  |  |
| Binocular | -0.07 ± 0.08 | -0.13 ± 0.12 | 0.05 |
| Right eye | -0.03 ± 0.07 | -0.10 ± 0.12 | ***0.02*** |
| Left eye | -0.04 ± 0.08 | -0.10 ± 0.09 | ***0.02*** |
| Visual acuity 25% contrast (logMAR) | |  |  |
| Binocular | 0.03 ± 0.11 | -0.03 ± 0.11 | 0.09 |
| Right eye | 0.08 ± 0.11 | 0.00 ± 0.11 | ***0.02*** |
| Left eye | 0.09 ± 0.13 | 0.02 ± 0.11 | 0.08 |
| Visual field mean deviation (dB) | |  |  |
| Right eye | -0.01 ± 1.96 | 0.85 ± 1.76 | 0.14 |
| Left eye | -0.15 ± 1.94 | 0.49 ± 1.68 | 0.25 |
| Retinal nerve fiber layer (µm) |  |  |  |
| Right eye | 81.6 ± 12.2 | 87.7 ± 8.8 | 0.06 |
| Left eye | 79.8 ± 8.9 | 86.3 ± 10.4 | ***0.03*** |
| Average cup-to-disc ratio |  |  |  |
| Right eye | 0.67 ± 0.07 | 0.49 ± 0.11 | ***0.000*** |
| Left eye | 0.66 ± 0.10 | 0.49 ± 0.09 | ***0.000*** |
| Vertical cup-to-disc ratio |  |  |  |
| Right eye | 0.67 ± 0.07 | 0.47 ± 0.10 | ***0.000*** |
| Left eye | 0.66 ± 0.11 | 0.49 ± 0.11 | ***0.000*** |
| MoCA cognitive test | 28.2 ± 1.4 | 28.1 ± 1.9 | 0.88 |

***Failure rates of travelling wave transmission***

In order to evaluate the failure rates of traveling wave transmission, we calculated the Intra/Inter Ratio: a value greater than 1 means that the failure rate for the intra-hemispheric condition is greater than that of the inter-hemispheric condition, a value equal to 1 means the failure rates for the two conditions are the same, and a value smaller than 1 means that the failure rate for the intra-hemispheric condition is lower than that of the inter-hemispheric condition. The Intra/Inter Ratio of the glaucoma group (mean = 1.0 ± 0.3) was significantly higher than that of the control group (mean = 0.8 ± 0.4), independent-samples t-test t(43) = 2.9, p = 0.006.

We further analyzed the failure rates with a 2 (Conditions: intra-, inter-) x 2 (Group: control, glaucoma) mixed factorial ANOVA. Although the failure rates were reported as percentages (i.e., percentage of trials that did not elicit a response), we treated these values as parametric data. There were a significant Condition main effect F(1, 43) = 7.9, p = 0.007, partial η^2^ = 0.16 and a significant Condition x Group interaction effect F(1, 43) = 6.9, p = 0.01, partial η^2^ = 0.14. Overall, the inter- failure rate was significantly higher than the intra- failure rate, but pairwise comparisons showed that these rates were not different from each other for glaucoma group, p = 0.9; however, the inter- was significantly larger than the intra- failure rate for the control group, p < 0.001. In addition, the intra-hemispheric failure rate was significantly higher for the glaucoma group than for the control group, p = 0.014, but the inter-hemispheric failure rate was similar for the two groups, p = 0.42. Averages for the intra- and inter-hemispheric traveling wave transmission failure rates and the Intra/Inter Ratio for the two groups are shown in Table 2 and in Figure 3.

Table 2. Averages (± SD) for intra- and inter-hemispheric traveling wave transmission failure rates and the Intra/Inter Ratio for the control and glaucoma group.

| Group | Intra-hemispheric failure rate (%) | Inter-hemispheric failure rate (%) | Intra/Inter Ratio |
| --- | --- | --- | --- |
| Control | 49 ± 3 | 64 ± 3 | 0.8 ± 0.4 |
| Glaucoma | 70 ± 2 | 70 ± 2 | 1.0 ± 0.3 |


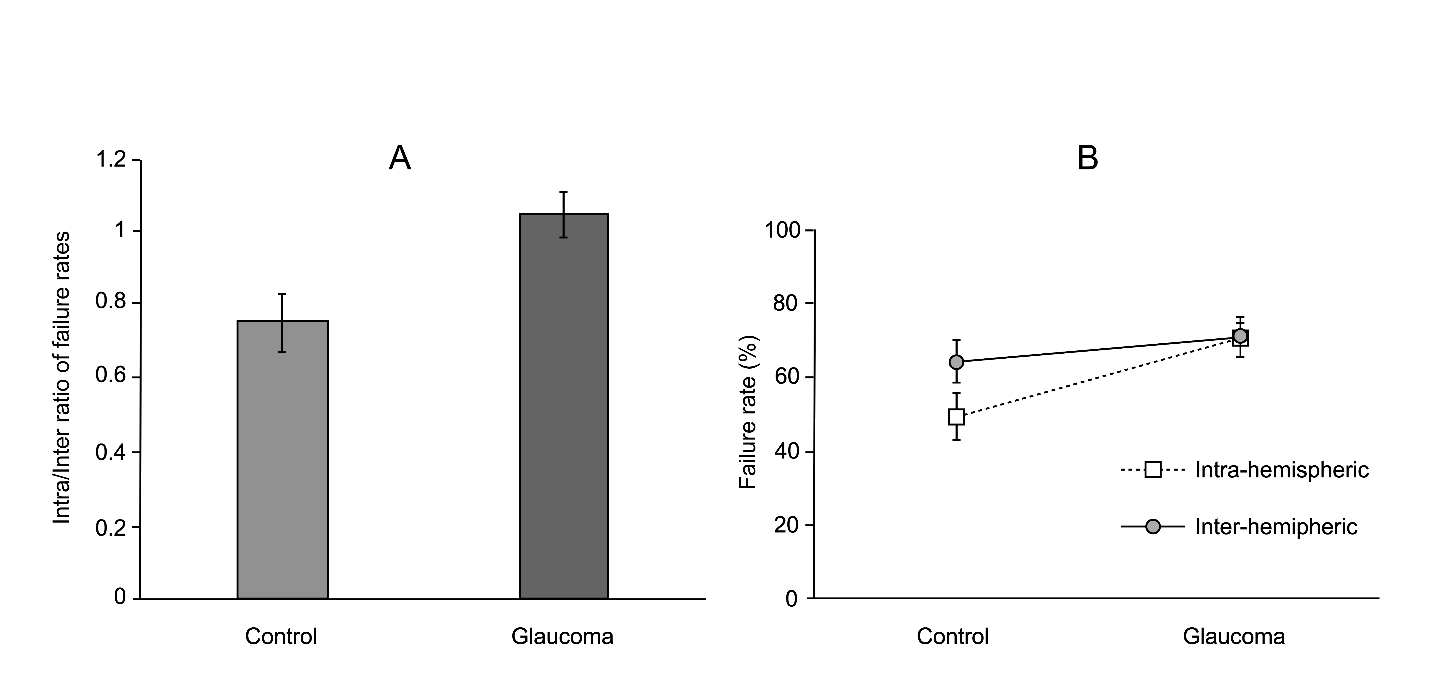
Figure 3. Traveling wave transmission failure rates of in the intra- and inter- hemispheric conditions, for the control and glaucoma group. Panels show: A) intra-/inter-hemispheric failure rate ratio (Intra/Inter Ratio); and B) average failure rates. Error bars are ± 1SE.

***TW propagation time***

The Intra/Inter Ratio for travelling wave propagation time had a median of 1.3 (mean = 1.6 ± 1.2) for the glaucoma group and a median of 0.93 (mean = 0.87 ± 0.2) for the control group. Because of high variability in the data, the difference between groups was assessed with a non-parametric test. The Mann-Whitney U test showed that the two groups differed significantly U = 55, p < 0.001. These results indicate not only a significant difference between the two groups, but also that the time of the travelling wave propagation was shorter for the inter- than for the intra-hemispheric condition for the glaucoma group (Intra/Inter Ratio greater than 1), while the opposite was true for the control group (Intra/Inter Ratio smaller than 1). The results are shown in Figure 4 panel A. These results were confirmed with an independent-samples t-test on the log transformed data, t(36) = 3.77, p = 0.001.

We also computed the time difference of the travelling wave propagation for the two conditions (inter - intra). For the control group, there was a time penalty for wave propagation in the inter-hemispheric condition of a median of 0.26s (mean = 0.49 ± 0.70s). For the glaucoma group, the median difference was -0.52s (mean = -0.59 ± 1.1s), which also indicates that the propagation was faster in the inter- than in the intra- hemispheric condition. The Mann-Whitney U test showed that the two groups differed significantly U = 60, p = 0.001 on this measure as well. This result is shown in Figure 4 panel B.

Travelling wave propagation time was further analyzed with a 2 (Conditions: intra-, inter) x 2 (Group: control, glaucoma) mixed factorial ANOVA. This analysis revealed only a significant Condition x Group interaction effect, F(1,36) = 13.7, p = 0.001, partial η^2^ = 0.28. Follow-up analysis showed that the propagation time was significantly longer for the inter- than for the intra-hemispheric condition for the control group p = 0.014, while the opposite was true for the glaucoma group, p = 0.012. The results are shown in Figure 4 panel C.


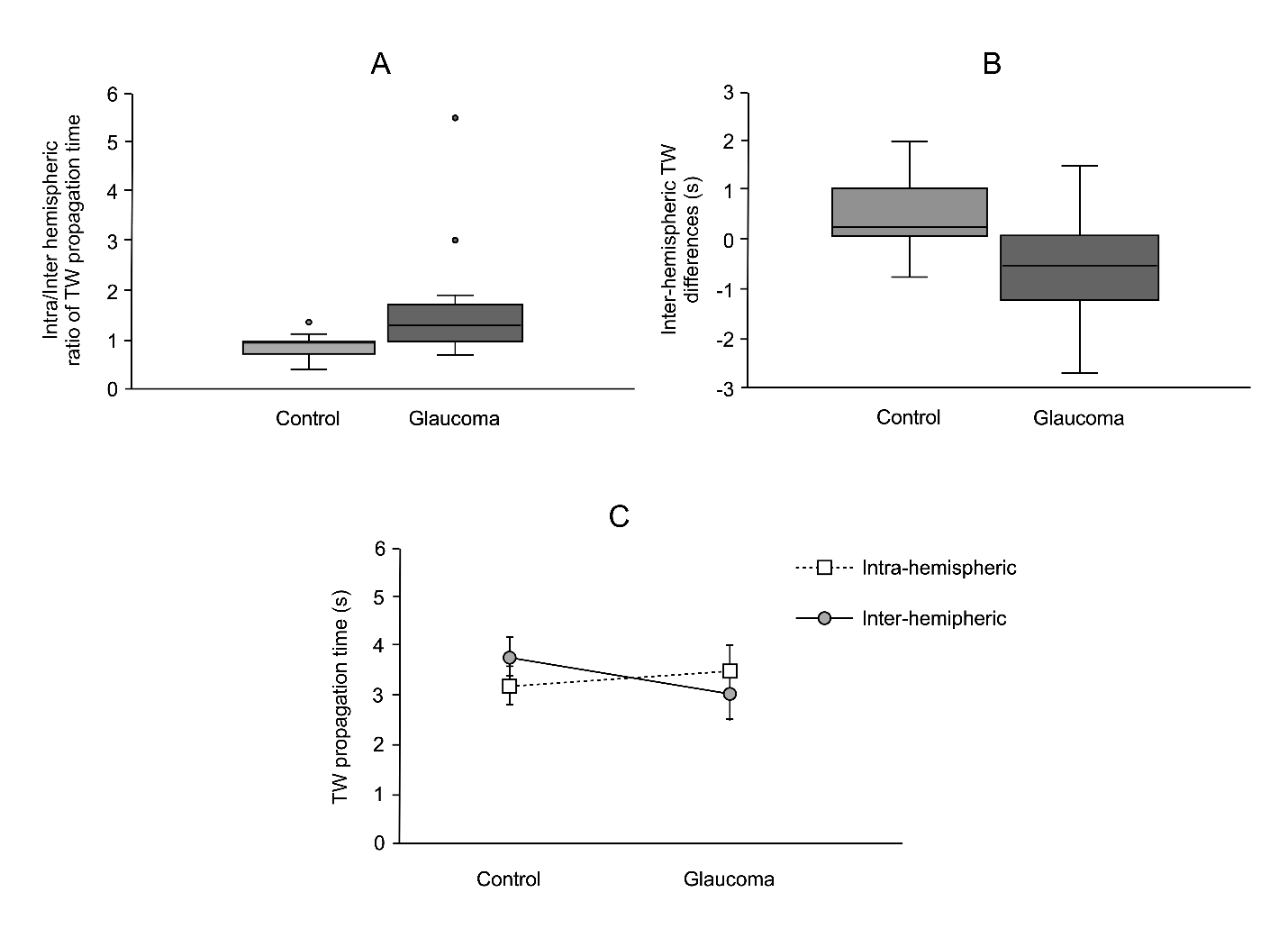


Figure 4. Traveling wave propagation time in the inter- and intra- hemispheric conditions for the two groups. Panels show: A) the Intra/Inter Ratios where a value smaller than 1 indicates faster propagation time in the intra- than in the inter-hemispheric conditions; B) Time difference between the two conditions, and C) the average time for traveling wave propagation. Error bars are ± 1SE.

***Travelling wave: POAG vs NTG***

In the glaucoma group, 14 patients (mean age: 66 ± 11 years) had a diagnosis of POAG and 6 patients (mean age: 63 ± 14 years) had NTG. The 2 subgroups were matched in age (p = 0.59). These 2 subgroups did not differ significantly in any psychophysical measures: intra- and inter-hemispheric failure rates of travelling wave transmission, the Intra/Inter Ratio of the failure rates, intra- and inter-hemispheric travelling wave propagation time, the Intra/Inter Ratio of the propagation time, and the time difference in travelling wave propagation between intra- and inter-hemispheric conditions (smallest p = 0.16).

**Discussion**

In this study we examined travelling wave dynamics of binocular rivalry in patients with mild glaucoma who otherwise had no significant functional deficits. Wave transmission failure rates were equally high for the inter- and intra- hemispheric conditions in the glaucoma group, but the control participants had a significantly lower failure rate for the intra- than for the inter-hemispheric condition. Interestingly, wave propagation was faster for the inter- than for the intra- hemispheric condition in the glaucoma group, while the opposite was true for the control group. These results indicate strong intra-hemispheric inhibitory processes in the visual cortex and an excitability of the callosal transfer in mild glaucoma.

During binocular rivalry with traditional stimuli two different images viewed dichoptically compete for perceptual dominance, resulting in a bistable percept whose rate of change and time of single stimulus dominance can be quantified (Lee et al., 2007; Blake & Wilson, 2011). These measures provide information about excitatory-inhibitory neural activity both within and between hemispheres and reveal functional aspects of the corpus callosum — the main structure involved in inter-hemispheric transfer (Berlucchi, 2014). The Wilson et al.’s (2001) travelling wave paradigm is a special case of binocular rivalry that is suitable for studying the gradual change of perceptual dominance, thus providing further insights into the neural dynamics of binocular rivalry and the integrity of inter-hemispheric transfer.

The travelling wave paradigm involves presenting a low contrast stimulus to one eye and a high contrast stimulus to the other eye that typically dominates continuously. This is in accordance to the Levelt’s first law of binocular rivalry which states that increasing the strength of the stimulus in one eye will result in a prolonged dominance of this stimulus (Levelt, 1966). Nevertheless, when a local increment of high contrast is presented briefly on the low contrast stimulus, this can release the strong inhibition allowing the suppressed stimulus to start a wave of dominance with its origin at the trigger’s location. We found that travelling wave transmission failure rate of the intra-hemispheric condition was significantly higher in mild glaucoma than in controls, suggesting stronger than normal inhibitory actions of intra-hemispheric processing. Rivalry dynamics may depend on the balance between the excitatory (i.e., glutamate) and inhibitory (i.e., γ-aminobutyric acid, GABA) neurotransmitters in the visual cortex. Because a relationship with visual performance has been found only for GABA but not for glutamate level in the occipital cortex (Kurcyus et al., 2018), our results may be explained by an upregulation of the GABA-mediated inhibitory actions in the visual cortex in mild glaucoma. This is supported by the fact that there is a genetic component of glaucoma associated with GABA metabolism dysregulation that is common both to POAG and NTG (Bailey et al., 2014). However, this inhibitory neural activity is not strong enough to be detected with traditional rivalry paradigm using stimuli that are equal in strength, because our lab has shown recently that the intra-hemispheric rivalry rate (i.e., the rate of perceptual switches in dominance) is not affected in these patients (Samet et al., 2019).

Wilson et al. (2001) showed that the travelling wave of dominance propagate across V1 at a cortical speed predicted by the cortical magnification factor, and, in conditions where inter-hemispheric transfer of visual information is involved, there is a time penalty likely due to the neural transmission going through the long callosal fibers. This delay of transfer between hemispheres was initially measured in 4 experienced observers (Wilson et al., 2001), but the finding was later replicated although high variability in the data was observed (Genç et al., 2011; Spiegel et al., 2015). The control group in our study showed a median delay of 260 ms in inter-hemispheric wave propagation and, despite highly variable data, the vast majority of the participants (19 out of 25) showed longer inter- than intra-hemispheric propagation time.

Interestingly, the glaucoma group produced the opposite effect: there was an overall faster inter-hemispheric propagation, with only 5 out of 20 patients showing the delay observed in controls. These results are consistent with data from patients with mild traumatic brain injury who are susceptible to long axonal injury (Spiegel et al., 2015). It has been shown that in patients with mild glaucoma — as were those included in this study — there is a volumetric gain in some brain structures including the corpus callosum, which becomes atrophic due to neurodegeneration only in moderate and advanced stages (Williams et al., 2013, Boucard et al., 2016). The increase in volume of the corpus callosum in the initial stages of the disease may be indicative of inflammatory responses to neuronal injury (Streit & Xue, 2012) that can produce an increase in neuronal excitability (Galic et al., 2012; Goldstein et al., 2016) in the long callosal axonal fibers, resulting in faster inter-hemispheric transfer. The chronic inflammatory responses later lead to neurodegeneration as seen in more advanced stages of disease. Alternatively, increased volume in neural structures could imply cortical plasticity and improved neural function (Williams et al., 2013), but this explanation is unlikely given that faster performance was selectively found only for the inter-hemispheric condition. Nevertheless, faster inter- than intra-hemispheric wave propagation has been reported in two neuro-injured clinical populations, both in mild stages, one chronic (i.e., glaucoma) and one acute (i.e., traumatic brain injury) (Spiegel et al., 2015). More research involving imaging techniques is needed to elucidate these findings.

In conclusion, travelling wave dynamics of binocular rivalry dominance are abnormal in patients with mild glaucoma irrespective of type (i.e., NTG or POAG). The high intra-hemispheric failure rate of wave transmission suggests strong inhibitory processes in the visual cortex that are probably GABAergic mediated. Faster inter- than intra-hemispheric wave propagation implies excitability of the neural transfer between the two hemispheres possibly due to inflammatory responses to neural injury in the corpus callosum. Although this latter finding is counterintuitive, it is consistent with results from mild traumatic brain injury (Spiegel et al., 2015) and with imaging findings of greater than normal volume of the corpus callosum in early stages of glaucoma (Williams et al., 2013). These results suggest degenerative mechanism in glaucoma that can be detected behaviorally before significant functional deficits.

**Acknowledgments**

This work was supported by the BrightFocus Foundation (Grant # G2017093). The authors thank Dr. Hugh Wilson for helpful discussions during the design phase of the study. The authors also thank all the participants who took part in this study.

**References**

Alais, D. & Blake, R. (1999). Grouping visual features during binocular rivalry. *Vision Research,* 39, 4341–4353.

Bailey, J.N., Yaspan, B.L., Pasquale, L.R., Hauser, M.A., Kang, J.H., Loomis, S.J., et al. (2014). Hypothesis-independent pathway analysis implicates GABA and acetyl-CoA metabolism in primary open-angle glaucoma and normal-pressure glaucoma. *Human Genetics,* 133, 1319–1330.

Berlucchi, G. (2014). Visual interhemispheric communication and callosal connections of the occipital lobes. *Cortex,* 56, 1–13.

Black, A.A., Wood, J.M. & Lovie-Kitchin, J.E. (2011). Inferior field loss increases rate of falls in older adults with glaucoma. *Optometry and Vision Science,* 88, 1275–1282.

Blake, R. & Wilson, H.R. (2011). Binocular vision. *Vision Research,* 51, 754–770.

Blake, R., O'Shea, R.P. & Mueller, T.J. (1992). Spatial zones of binocular rivalry in central and peripheral vision. *Visual Neuroscience,* 8, 469–478.

Boucard, C.C., Hanekamp, S., Ćurčić-Blake, B., Ida, M., Yoshida, M. & Cornelissen, F.W. (2016). Neurodegeneration beyond the primary visual pathways in a population with a high incidence of normal-pressure glaucoma. *Ophthalmic Physiological Optics,* 36, 344–353.

Brin, T.A., Tarita-Nistor, L., González, E.G., Trope, G.E. & Steinbach, M.J. (2019). Vection responses in early glaucoma. *Journal of Glaucoma,* 28, 68–74.

Bullimore, M.A., Wood, J.M. & Swenson, K. (1993). Motion percetion in glaucoma. *Investigative Ophthalmology & Visual Science,* 34, 3526–3533.

Chen, W.W., Wang, N., Cai, S., Fang, Z., Yu, M., Wu, Q., et al., (2013). Structural brain abnormalities in patients with primary open-angle glaucoma: a study with 3T MR imaging. *Investigative Ophthalmology & Visual Science,* 54, 545–554.

Essock, E.A., Fechtner, R.D., Zimmerman, T.J., Krebs, W.K. & Nussdorf, J.D. (1996). Binocular function in early glaucoma. *Journal of Glaucoma,* 5, 395–405.

Friedman, D.S., Feeman, E., Munoz, B., Jampel, H.D. & West, S.K. (2007). Glaucoma and mobility performance. *Ophthalmology,* 114, 2232–2237.

Galic, M.A., Riazi, K. & Pittman, Q.J. (2012). Cytokines and brain excitability. *Frontiers in Neuroendocrinology,* 33, 116–125.

Garaci, F.G., Bolacchi, F., Cerulli, A., Melis, M., Spanò, A., Cedrone, C., et al., (2009). Optic nerve and optic radiation neurodegeneration in patients with glaucoma: in vivo analysis with 3-T diffusion-tensor MR imaging. *Radiology,* 252, 496–501.

Genç, E., Bergmann, J., Tong, F., Blake, R., Singer, W. & Kohler A. (2011). Callosal connections of primary visual cortex predict the spatial spreading of binocular rivalry across the visual hemifields. *Frontiers in Human Neuroscience,* 5, 161doi: 10.3389/fnhum.2011.00161

Goldstein, E.Z., Church, J.S., Hesp, Z.C., Popovich, P.G. & Mctigue, D.M. (2016). A silver lining of neuroinflammation: Beneficial effects on myelination. *Experimental Neurology,* 283, 550–559.

Gupta, N., Ang, L.-C., Noel De Tilly, L., Bidaisee, L. & Yucel, Y.H. (2006). Human glaucoma and neural degeneration in intracranial optic nerve, lateral geniculate nucleus, and visual cortex. *British Journal of Ophthalmology,* 90, 674–678.

Hawkins, A.S., Szlyk, J.P., Ardickas, Z., Alexander, K.R. & Wilsensky, J.T. (2003). Comparison of contrast sensitivity, visual acuity, and Humphrey visual field testing in patients with glaucoma. *Journal of Glaucoma,* 12, 134–138.

Haymes, S.A., Leblanc, R.P., Nicolela, M.T., Chiasson, L.A. & Chauhan, B.C. (2007). Risk of falls and motor vehicle collisions in glaucoma. *Investigative Ophthalmology & Visual Science,* 48, 1149–1155.

Hernowo, A.T., Boucard, C.C., Jansonius, N.M., Hooymans, J.M. & Cornelissen, F.W. (2011). Automated morphometry of the visual pathway in primary open-angle glaucoma. *Investigative Ophthalmology & Visual Science,* 52, 2758–2766.

Kanjee, R., Yucel, Y.H., Steinbach, M.J., Gonzalez, E.G. & Gupta, N. (2012). Delayed saccadic eye movements in glaucoma. *Eye and Brain,* 4, 63–68.

Kotecha, A., O’Leary, N., Melmoth, D., Gant, S. & Crabb, D.P. (2009). The functional consequences of glaucoma for eye-hand coordination. *Investigative Ophthalmology & Visual Science,* 50, 203–213.

Kurcyus, K., Annac, E., Hanning, N.M., Harris, A.D., Oeltzschner, G., Edden, R. & Riedl, V. (2018). Opposite dynamics of GABA and glutamate levels in the occipital cortex during visual processing. *Journal of Neuroscience,* 38, 9967–9976.

Lamirel, C., Milea, D., Cochereau, I., Duong, M.H. & Lorenceau, J. (2014). Impaired saccadic eye movement in primary open-angle glaucoma. *Journal of Glaucoma,* 23, 23–32.

Lee, S.H. & Blake, R. (1999). Rival ideas about binocular rivalry. *Vision Research,* 39, 1447–1454.

Lee, S.H., Blake, R. & Heeger, D.J. (2007). Hierarchy of cortical responses underlying binocular rivalry. *Nature Neuroscience,* 10, 1048–1054.

Levelt, W.J.M. (1966). The alternation process in binocular rivalry. *British Journal of Psychology,* 57, 225–238.

McKendrick, A.M., Badcock, D.R. & Morgan, W.H. (2005). The detection of both global motion and global form is disrupted in glaucoma. *Investigative Ophthalmology & Visual Science,* 46, 3693–3701.

McKendrick, A.M., Sampson, G.P., Walland, M.J. & Badcock D.R. (2007). Contrast sensitivity changes due to glaucoma and normal aging: low-spatial-frequency losses in both magnocellular and parvocellular pathways. *Investigative Ophthalmology & Visual Science,* 48, 2115–2122.

Miller, S.M., Liu, G.B., Ngo, T.T., Hooper, G., Riek, S., Carson, R.G. & Pettigrew, J.D. (2000). Interhemispheric switching mediates perceptual rivalry. *Current Biology,* 10, 383–392.

O'Shea, R.P. & Corballis, P.M. (2003). Binocular rivalry in split-brain observers. *Journal of Vision,* 3, 610–615.

Quigley, H.A. & Broman, A.T. (2006). The number of people with glaucoma worldwide in 2010 and 2020. *British Journal of Ophthalmology,* 90, 262–267.

Quigley, H.A. (1999). Neural death in glaucoma. *Progress in Retinal and Eye Research,* 18, 39–57.

Ramulu, P. (2009). Glaucoma and disability: which tasks are affected, and at what stage of disease? *Current Opinion in Ophthalmology,* 20, 92–98.

Ramulu, P.Y., Swenor, B.K., Jefferys, J.L., Friedman, D.S. & Rubin, G.S. (2013). Difficulty with out-loud and silent reading in glaucoma. *Investigative Ophthalmology & Visual Science,* 54, 666–672.

Ramulu, P.Y., West, S.K., Munoz, B., Jampel, H.D. & Friedman, D.S. (2009). Glaucoma and reading speed: The Salisbury Eye Evaluation Project. *Archives of Ophthalmology,* 127, 82–87.

Samet, S., González, E.G., Trope, G.E. & Tarita-Nistor, L. (2019). Intra- and inter-hemispheric processing during binocular rivalry in early glaucoma. *Investigative Ophthalmology & Visual Science,* E-abstract. In press.

Silverman, S.E., Trick, G.L. & Hart, W.M. (1990). Motion perception is abnormal in primary open-angle glaucoma nd ocular hypertension. *Investigative Ophthalmology & Visual Science,* 31, 722–729.

Spiegel, D.P., Laguë-Beauvais, M., Sharma, G. & Farivar R. (2015). Inter-hemispheric wave propagation failures in traumatic brain injury are indicative of callosal damage. *Vision Research,* 109, 38–44.

Streit, W.J. & Xue, Q.S. (2012). Alzheimer's disease, neuroprotection, and CNS immunosenescence. *Frontiers in Pharmacology,* 3, 138. eCollection.

Tarita-Nistor, L., Hadavi, S., Steinbach, M.J., Markowitz, S.N. & González, E.G. (2014). Vection in patients with glaucoma. *Optometry and Vision Science,* 91, 556–563.

Tong, F., Meng, M. & Blake, R. (2006). Neural bases of binocular rivalry. *Trends in Cognitive Sciences,* 10, 502–511.

Trick, G.L., Steinman, S.B. & Amyot, M. (1995). Motion perception deficits in glaucomatous optic neuropathy. *Vision Research,* 35, 2225–2233.

Turano, K.A., Rubin, G.S. & Quigley, H.A. (1999). Mobility performance in glaucoma. *Investigative Ophthalmology & Visual Science,* 40, 2803–2809.

Varma, R., Lee, P.P., Goldberg, I. & Kotak, S. (2011). An assessment of the health and economic burdens of glaucoma. *American Journal of Ophthalmology,* 152, 515–522.

Westcott, M.C., Fitzke, F.W. & Hitchings, R.A. (1998). Abnormal motion displacement thresholds are associated with fine scale luminance sensitivity loss in glaucoma. *Vision Research,* 38, 3171–3180.

Williams, A.L., Lackey, J., Wizov, S.S., Chia, T.M., Gatla, S., Moster, M.L., et al. (2013). Evidence for widespread structural brain changes in glaucoma: a preliminary voxel-based MRI study. *Investigative Ophthalmology & Visual Science,* 54, 5880–5887.

Wilson, H.R., Blake, R. & Lee, S.H. (2001). Dynamics of travelling waves in visual perception. *Nature,* 412, 907–910.

Zhang, Y.Q., Li, J., Xu, L., Zhang, L., Wang, Z.C., Yang, et al. (2012). Anterior visual pathway assessment by magnetic resonance imaging in normal-pressure glaucoma. *Acta Ophthalmologica,* 90, e295–302.
